# Cannabinoid Compounds as a Pharmacotherapeutic Option for the Treatment of Non-Cancer Skin Diseases

**DOI:** 10.3390/cells11244102

**Published:** 2022-12-16

**Authors:** Robert Ramer, Burkhard Hinz

**Affiliations:** Institute of Pharmacology and Toxicology, Rostock University Medical Centre, Schillingallee 70, D-18057 Rostock, Germany

**Keywords:** cannabinoids, cannabinoid receptors, endocannabinoid system, atopic dermatitis, allergic contact dermatitis, dermatomyositis, fibrosis, inflammation, pruritus, psoriasis, systemic sclerosis, wound healing

## Abstract

The endocannabinoid system has been shown to be involved in various skin functions, such as melanogenesis and the maintenance of redox balance in skin cells exposed to UV radiation, as well as barrier functions, sebaceous gland activity, wound healing and the skin’s immune response. In addition to the potential use of cannabinoids in the treatment and prevention of skin cancer, cannabinoid compounds and derivatives are of interest as potential systemic and topical applications for the treatment of various inflammatory, fibrotic and pruritic skin conditions. In this context, cannabinoid compounds have been successfully tested as a therapeutic option for the treatment of androgenetic alopecia, atopic and seborrhoeic dermatitis, dermatomyositis, asteatotic and atopic eczema, uraemic pruritis, scalp psoriasis, systemic sclerosis and venous leg ulcers. This review provides an insight into the current literature on cannabinoid compounds as potential medicines for the treatment of skin diseases.

## 1. Introduction

Numerous studies have shown that the use of cannabinoids to treat skin diseases has potential benefits for patients. In a recent review paper, we pointed out the effects of cannabinoid substances on malignant changes of the skin such as melanoma and squamous cell carcinoma [1]. Many publications in recent years suggest that cannabinoids could also play a useful role for patients as systemic and topical applications in inflammatory, allergic, fibrotic and pruritic skin diseases as well as in skin care. For this reason, these effects could be of particular importance to dermatologists. The use of cannabinoids in the context of skin diseases has continued to gain attention with increasing commercial interest [2].

The prominent role of the endocannabinoid system in skin homeostasis was demonstrated in a comprehensive study in 2007, which found a strong influence of cannabinoid receptors on the pathogenesis of allergic contact dermatitis [3]. This pioneering study led to an avalanche of publications on the role of cannabinoid receptors in the skin, the regulation of cannabinoid-triggered receptors in pathophysiological conditions of the skin, and the possibility of testing cannabinoid-based drugs for numerous skin diseases. Meanwhile, the endocannabinoid system has been implicated in various physiological processes of the skin, such as melanogenesis, maintenance of redox balance in response to ultraviolet (UV) radiation, wound healing, barrier functions, control of immunological sensitivity, sebaceous gland functions and hair growth. Due to the multiple effects of cannabinoid-activated receptors in the skin, the cutaneous endocannabinoid system was appropriately termed the “c(ut)annabinoid” system in a recent comprehensive review of cannabinoid effects in the skin [4].

**Figure 1:**
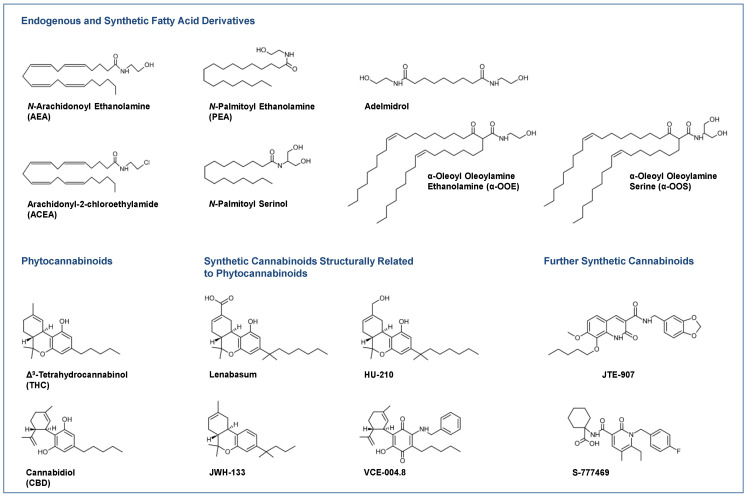
Selected endocannabinoids and phytocannabinoids and their derivatives currently under investigation or in use for the treatment of skin diseases.

Skin conditions where the efficacy of different cannabinoids has been successfully tested in in vitro and in vivo models include atopic dermatitis [5], psoriasis [6,7] and dermatomyositis [8], an idiopathic inflammatory myopathy. In the latter report, the cannabinoid receptor 2 (CB_2_) agonist lenabasum (Figure 1), a 9-carbon 1,1-dimethylheptyl side-chain analogue of the tetrahydrocannabinol (THC) metabolite THC-11-oic acid (also known as JBT-101, ajulemic acid, formerly anabasum), was discovered as a potential treatment for dermatomyositis, which has since been designated as an orphan drug by the European Medicines Agency (EMA) [9]. Moreover, the same cannabinoid was granted orphan drug designation by the EMA for the treatment of systemic sclerosis (scleroderma) as a typical fibrotic disease [10]. Particularly in connection with research into the antifibrotic properties of cannabinoids, a trend in drug design is already emerging towards cannabinoid substances that, chemically modified, interact with other receptors in addition to the classic cannabinoid targets or are specially formulated. This is especially evident with the non-intoxicating phytocannabinoid cannabidiol (CBD; Figure 1) [11], the antifibrotic CBD aminoquinone VCE-004.8 (Figure 1), a dual receptor agonist at the CB_2_ receptor and peroxisome proliferation-activated receptor γ (PPARγ) [12,13], and EHP-101, a lipidic formulation of VCE-004.8 [14,15]. 

Most clinical studies have focused on the effects of CBD in various dosage forms and *N*-palmitoyl ethanolamine (PEA; Figure 1) for symptom relief in atopic dermatitis [16,17,18] and other diseases. For some diseases, there are only case reports on the potential efficacy of cannabinoids, such as in epidermolysis bullosa [19,20], pyoderma gangrenosum [21,22], pruritus due to cholestatic liver disease [23] and lichen simplex chronicus [24]. Figure 1 shows a collection of chemical formulae of the endocannabinoids, endocannabinoid-like substances and phytocannabinoids and their derivatives that are currently being studied or used for the treatment of skin diseases.

The aim of the present review is to summarise the currently available knowledge on the effect of cannabinoids on the pathogenesis of various selected skin diseases in the context of the respective existing pharmacotherapy. The presentations of the pharmacotherapeutic options currently used for the individual indications provide an insight into the drugs competing with cannabinoids and are intended to further identify potential combination partners in cannabinoid therapy that may be able to achieve synergistic effects in upcoming clinical trials. To this end, the components of the endocannabinoid system are first described in general terms, and then their distribution and function in the skin and their regulation under pathophysiological conditions are outlined. Finally, the individual preclinical and clinical results of the meanwhile numerous studies on the effects of cannabinoids in skin diseases are presented.

## 2. The Endocannabinoid System: A Brief Overview

The endocannabinoid system includes the endocannabinoid receptors, their endogenous agonists and the enzymes that synthesise and degrade the endocannabinoids. 

### 2.1. Classic Cannabinoid Receptors

Long before the discovery of cannabinoid receptors, in the year 1978, Mechoulam and Carlini wrote “We believe that cannabinoids act on specific receptors.” [25]. However, that was more than a decade before the first cannabinoid receptor was actually cloned. Thus, the cannabinoid receptors, CB_1_ and CB_2_, a class of heptahelical pertussis toxin-sensitive G_i/o_ protein-coupled membrane receptors were discovered in the early 1990s [26,27]. Δ^9^-tetrahydrocannabinol (THC; Figure 1), the major psychoactive constituent of *Cannabis sativa* L. has been shown to act as a full agonist at the CB_2_ receptor and as a partial agonist at the CB_1_ receptor and can therefore be classified as a phytocannabinoid. In contrast, another major constituent of cannabis, the non-intoxicating CBD, exhibits a much weaker affinity for cannabinoid receptors [28] and has been reported to act as a negative allosteric modulator of the CB_1_ receptor [29]. The complex mechanisms of action and the underlying interference with various receptors on which CBD exerts its effects are discussed below.

### 2.2. Endocannabinoids

**Figure 2:**
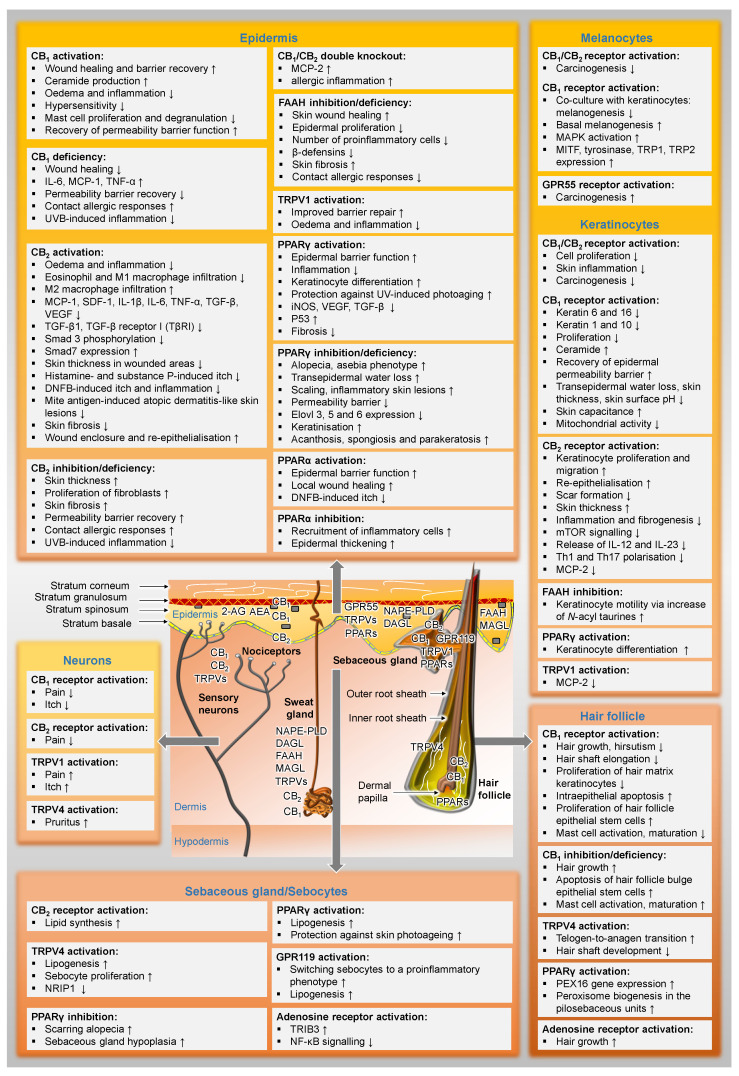
Distribution of the elements of the endocannabinoid system in the skin and their functional effects. In the text boxes, the regulations proven in the literature have been indicated. The term *deficiency* always refers to the fact that results from experiments with knockout animals are included here. ↑, upregulated; ↓, downregulated; AEA, *N*-arachidonoyl ethanolamine (anandamide); 2-AG, 2-arachidonoyl glycerol; CB_1_, cannabinoid receptor 1; CB_2_, cannabinoid receptor 2; DAGL, diacylglycerol lipase; DNFB, 1-fluoro-2,4-dinitrobenzene; Elovl 3, 5 and 6, elongation of very-long-chain fatty acids-like group of enzymes; GPR, G protein-coupled receptor; FAAH, fatty acid amide hydrolase; IL, interleukin; iNOS, inducible nitric oxide synthase; MAGL, monoacylglycerol lipase; MAPK, mitogen-activated protein kinases; MCP, monocyte chemoattractant protein; MITF, microphthalmus-associated transcription factor; mTOR, mammalian target of rapamycin; NAPE-PLD, *N*-acylphosphatidylethanolamine-hydrolysing phospholipase D; NF-κB, nuclear factor ‘kappa-light-chain-enhancer’ of activated B-cells; NRIP1, nuclear receptor interacting protein-1; PEX16, peroxin 16; PPAR, peroxisome proliferator-activated receptor; SDF-1, stromal cell-derived factor-1; Smad, small mothers against decapentaplegic homolog; TGF-β, transforming growth factor-β; TNF-α, tumour necrosis factor-α; TRIB3, tribbles homolog 3; TRP, tyrosinase-related protein; TRPV, transient receptor potential vanilloid; UVB, ultraviolet B radiation; VEGF, vascular endothelial growth factor.

*N*-arachidonoyl ethanolamine (AEA, anandamide; Figure 1) and 2-arachidonoyl glycerol (2-AG) were the first arachidonic acid derivatives described as endogenous agonists at cannabinoid receptors [30,31]. Concerning receptor interaction modes, AEA was found to be a partial agonist at the CB_1_ receptor with an affinity comparable to THC [32,33] and a weak agonist at the CB_2_ receptor [34], whereas 2-AG exhibits full agonistic properties at the CB_2_ receptor [33,34]. 

As early as 1998, it was hypothesised that dopamine molecules are packaged and maintained as *N*-acyl dopamines in the dense nuclear vesicles of the chemoreceptor cells of the carotid body, with arachidonic acid as a possible cis-unsaturated fatty acid forming the acyl backbone [35]. From this group of initially still hypothetical endogenous substances, *N*-arachidonoyl dopamine (NADA) was synthesised in 2000 and investigated to what extent it shares with AEA the same transport and degradation mechanisms as well as properties for binding to cannabinoid receptors. Hereby, it was found that NADA does not bind to the corresponding dopamine receptors, but has a high affinity to the cannabinoid receptor CB_1_ [36]. Later, NADA was isolated from mammalian brain tissue, with the highest concentrations found in the striatum, hippocampus and cerebellum [37]. Around this time, 2-arachidonoyl glyceryl ether (2-AGE, noladin ether), a structural ether analogue of 2-AG with higher stability than 2-AG that preferentially binds to the CB_1_ receptor, was also discovered in porcine brain [38]. Another lipid of the endocannabinoid system is *O*-arachidonoyl ethanolamine (virodhamine), an ester derivative of arachidonic acid and ethanolamine, which has been detected in rat brain and human hippocampus and acts as an antagonist at the CB_1_ receptor and full agonist at the CB_2_ receptor [39]. Finally, it was recently discovered that pentadecanoylcarnitine, an endogenous metabolite of the essential fatty acid pentadecanoic acid found in bottlenose dolphin serum, is a fully potent CB_1_ and CB_2_ receptor agonist [40]. However, the significance of this endocannabinoid for the human body has not yet been clarified.

In addition, “endocannabinoid-like substances” such as PEA, oleoylethanolamide (OEA), stearoylethanolamide (SEA) and linoleoylethanolamide (LEA) lack binding affinity to cannabinoid receptors but have the highest concentrations in the human brain, with PEA being the most abundant (50%), followed by OEA (23. 6%) and SEA (13.9%), while AEA and 2-AG account for only 7.7% and 4.8%, respectively [41]. As recently reviewed, the mechanism of action of these fatty acids includes interference with receptors of the extended endocannabinoid system, and they have been found to share the corresponding synthesis and degradation enzymes with endocannabinoids (for review see [42]). According to some studies, PEA can enhance the effect of AEA in the sense of an “entourage effect” by down-regulating the expression and activity of the AEA-degrading enzyme fatty acid amide hydrolase (FAAH) [43] and causing a probably positive allosteric modulation of the AEA target transient receptor potential vanilloid 1 (TRPV1) [44]. Both endocannabinoid-degrading enzymes and other cannabinoid targets are discussed in more detail in the following chapters. As with PEA, the endocannabinoid-like compound SEA has been shown to enhance the effects of AEA by inhibiting FAAH [45]. A similar effect has also been described for 2-AG derivatives such as linoleoyl glycerol and palmitoyl glycerol, which likewise do not bind to cannabinoid receptors but act as entourage compounds for 2-AG by enhancing its effect at least partly by inhibiting inactivation of 2-AG [46]. 

### 2.3. Enzymes Involved in Biosynthesis and Degradation of Endocannabinoids

The enzymes responsible for the synthesis of endocannabinoids are, in the case of AEA, *N*-acylphosphatidylethanolamine-hydrolysing phospholipase D (NAPE-PLD) and, in the case of 2-AG, mainly phospholipase C or diacylglycerol lipases α and β (for review see [47]). The most important enzyme for the degradation of AEA is the already mentioned FAAH [48]. 2-AG is metabolised by two main pathways, namely via monoacylglycerol lipase (MAGL) or via the α/β-hydrolase domain (ABHD) containing enzymes ABHD6 and ABHD12 [49]. The endogenous hydrolysis and thus the biological inactivation of PEA is catalysed by the enzyme cysteine hydrolase *N*-acylethanolamine acid amidase (NAAA), which is mainly found in macrophages and B-lymphocytes [50,51].

### 2.4. Further Receptor Targets of Cannabinoids

Other receptor targets involved in cannabinoid action include ion channels of the transient receptor potential (TRP) family, such as TRPV1, which is activated, e.g., by AEA [52] and CBD [53]. Moreover, 2-AGE not only acts as a CB receptor agonist, but also has partial TRPV1 agonistic properties [38,54]. Furthermore, NADA has been described as a potent “endovanilloid”, i.e., an endogenous agonist at TRPV1 [37]. Likewise, the minor phytocannabinoids cannabigerol (CBG), cannabichromene (CBC), tetrahydrocannabivarin (THCV) and cannabigerolic acid (CBGA) were recently shown to stimulate TRPV1 [55]. TRPV2 was described as a target of CBD [56] and THC [57]. A number of phytocannabinoids were also tested for possible activation of TRPV3 and TRPV4. In these tests, CBD and THCV activated TRPV3-dependent Ca^2+^ currents, while cannabigerovarin (CBGV) and CBGA were able to sensitise TRPV3. Cannabidivarin (CBDV) and THCV stimulated TRPV4-mediated Ca^2+^ currents, while CBGA, CBGV, cannabinol (CBN) and CBG sensitised TRPV4 [58]. Finally, CBD and the minor phytocannabinoids CBC, CBN, CBDV, CBGV and THCV have been found to activate TRP channels of ankyrin type-1 (TRPA1) and the TRP channel of melastatin type-8 (TRPM8) [57]. 

A further target triggered by cannabinoid compounds is the G protein-coupled receptor 55 (GPR55), already discussed as a putative type 3 cannabinoid receptor [59], which can promote skin carcinogenesis [60]. GTPγS binding assays showed that GPR55 is coupled to Gα_13_ and mediates activation of the small G proteins ras homolog family member A (rhoA), cell division control protein 42 homolog (cdc42) and ras-related C3 botulinum toxin substrate 1 (rac1) [61]. In this pioneering study, GPR55 was found to be activated by AEA, 2-AG, 2-AGE, virodhamine, THC, abnormal CBD (abn-CBD; synthetic regioisomer of CBD), CP 55,940 (synthetic non-selective agonist with high affinity for CB_1_ and CB_2_ receptors) and PEA, but inhibited by CBD. The activating properties of THC and AEA were also confirmed in an investigation using the increase in intracellular calcium as an indicator of GPR55 activity [62]. Other cannabinoids with GPR55-activating properties here were the hydrolysis-stable AEA analogue *R*(+)-methanandamide and the aminoalkylindole JWH-015, which is known to be a specific CB_2_ agonist, while CP 55,940, 2-AG, PEA, virodhamine and abn-CBD did not significantly increase intracellular calcium levels [62]. Using a β-arrestin green fluorescent protein biosensor as readout of agonist-mediated receptor activation, a further study demonstrated that CP 55,940 acts as a GPR55 antagonist/partial agonist, while the CB_1_ receptor antagonists AM-251 and SR141716A (rimonabant) are GPR55 agonists [63]. As another G protein-coupled receptor, GPR119 was found to be activated by OEA [64]. 

Finally, there are several cannabinoids that have been shown to modulate GPR18, as summarised in a recent review [65]. In this context, it is interesting to note that it was already shown in 2006 that *N*-arachidonoyl glycine (NAGly; carboxylic metabolite of AEA) mediates effects such as an increase in calcium levels and a decrease in cAMP levels in GPR18-transfected cells compared to mock-transfected cells [66]. An GPR18-activating effect was later confirmed for *N*-palmitoyl glycine, an endogenous arachidonoyl amide, in the dorsal root ganglion cell line F11 [67]. In another report, NAGly was demonstrated to cause activation of mitogen-activated protein kinases (MAPK) p42/44 in GPR18-overexpressing HEK293 cells, as the most potent full agonist of a number of cannabinoids [68]. Other cannabinoids described as GPR18 full agonists in this study were abn-CBD, THC and AEA. The GPR18-mediated induction of MAPK activity was confirmed by other authors who focused on G-protein coupling of GPR18 and found that pertussis toxin completely blocked MAPK activity induced by abn-CBD and NAGly, but not by THC, demonstrating the involvement of multiple signal transduction pathways in GPR18 activation [69].

An important class of receptors triggered by cannabinoids is the group of peroxisome proliferator-activated receptors (PPAR), which are involved in skin inflammation, keratinocyte proliferation and differentiation, metabolism and oxidative stress response, as recently reviewed [70]. Of these, PPARα becomes activated by OEA [71] and PEA [72,73]. Furthermore, THC, although lacking binding affinity to PPARα, was found to upregulate and thereby enhance the transcriptional activity of PPARα [74]. Using luciferase reporter assays, another report demonstrated that PPARα transcriptional activity is increased by the non-selective cannabinoid receptor agonist WIN 55,212-2 as well as by AEA, OEA, virodhamine and 2-AGE [75]. PPARγ was found to be activated by THC [76] and CBD [77]. Further cannabinoids with PPARγ-activating properties include AEA, 2-AG and NADA, as well as the synthetic cannabinoids HU-210 (Figure 1), WIN 55,212-2, CP 55,940 (for review see [78]) and lenabasum [79]. Increased activity and intracellular concentration of PPARγ was also induced by THC and JWH-015 in another study [80]. Finally, cannabidiolic acid (CBDA) has been shown to cause abrogation of PPARβ/δ-related signalling [81] and THC has been found to cause PPARβ/δ-mediated suppression of PPARα function [82].

## 3. The Endocannabinoid System in the Skin

### 3.1. Distribution of Components of the Endocannabinoid System in the Skin

#### 3.1.1. Classic Endocannabinoid System

The presence of the endocannabinoid system in skin tissue was demonstrated in the mid-1990s by the discovery of the endocannabinoid AEA in rat skin, where it was found in concentrations similar to those in rat brain [83]. Later, endocannabinoids were shown to cause tonic activation of local cannabinoid receptors in rat skin [84]. Among others, AEA and 2-AG were also found in cells derived from sweat glands [85] and in the hair follicle [86].

Classic cannabinoid receptors were detected in many cell types of the skin, such as nerve fibre bundels of the skin, mast cells, macrophages, epidermal keratinocytes, epithelial cells of hair follicles, sebocytes and myoepithelial cells of the eccrine sweat glands [87]. Regarding the differential distribution of cannabinoid receptors in human skin tissue, Ständer et al. reported, that the CB_2_ receptor is expressed in basal keratinocytes, whereas CB_1_ receptors are present in keratinocytes of the stratum spinosum and granulosum, and that both cannabinoid receptors are detectable in nerve fibres of the epidermis, in small unmyelinated subepidermal nerves and in large myelinated nerves of the dermis [87]. Other skin structures with detectable CB_1_ receptor expression include differentiated sebaceous gland cells and epithelial cells of the infundibulum and inner root sheath of hair follicles, while CB_2_ receptors were found in undifferentiated cells of these niches. It is worth noting, however, that in another report the CB_2_ receptor could not be detected in human hair follicles by immunohistochemistry and quantitative RT-PCR [86]. The authors of the latter report detected CB_1_ receptors in the epithelium of human scalp hair follicles mainly in the outer root sheath, but not in the fibroblasts of the dermal papilla of the hair follicle. In a further study, cannabinoid receptors were detected in the sebaceous gland epithelium of normal human scalp sections [88]. In addition, both cannabinoid receptors were found in cells derived from sweat glands [85]. 

The enzymes that synthesise and degrade endocannabinoids (NAPE-PLD, DAGL, FAAH and MAGL) were also detected in human sweat glands [85]. A similarly complete endowment of the endocannabinoid system has also been demonstrated in normal human epidermal melanocytes [89]. Remarkably, that there are currently no data on the expression of enzymes that synthesise and degrade endocannabinoids in the hair follicle, although endocannabinoids have been detected here [86].

#### 3.1.2. Extended Endocannabinoid System 

Other cannabinoid-triggered receptors that have been detected in skin tissue are ion channels of the TRP family. For example, TRPV4 was found in the secretory cells of the eccrine sweat glands [90]. In addition to the two cannabinoid receptors, human keratinocytes have also been shown to express TRPV1 [55]. Moreover, TRPV3 was detected in the hair follicles [91]. Finally, TRPV1–4 but not TRPM8 and TRPA1 were found in a human sweat gland epithelial cell line [85]. 

Among PPARs, PPARα was found to be predominantly expressed in the epithelial compartment in basal keratinocytes and hair papilla cells [92]. Others detected PPARα, δ and γ in epidermis and hair follicle [93,94,95] as well as in the sebaceous gland [96]. Human keratinocytes have also been shown to express PPARα/γ/δ receptors and GPR55 [55]. In addition, PPARγ is present in primary cultured human sebocytes [97] as well as in fibroblasts from human skin biopsies [98]. Finally, GPR119 was detected in the sebaceous gland [99].

Figure 2 gives an overview of the distribution of cannabinoid-influenced receptors in the skin and already presents selected functional effects of these receptor activations or inhibitions in preparation for the following chapters.

### 3.2. The Endocannabinoid System as a Regulator of Skin Homeostasis

#### 3.2.1. Influence on Melanogenesis

As melanogenesis plays a crucial role in protecting the skin from UV radiation and oxidative stress, a recent study focused on cannabinoids as a protective pharmacotherapeutic option for the treatment of skin diseases caused by external factors. Here, CBD was found to stimulate melanogenesis via CB_1_ receptors by activating p38 and p42/44 MAPK and subsequently increasing the expression of microphthalmus-associated transcription factor (MITF), tyrosinase, tyrosinase-related protein 1 and 2 (TRP1, TRP2), all of which are involved in melanogenesis [100]. On the other hand, the CB_1_ agonist arachidonyl-2-chloroethylamide (ACEA; Figure 1) was shown to decrease melanogenesis in the melanoma cell line SK-Mel-1 co-cultured with the keratinocyte cell line HaCat under both basal and UVB irradiation stimulated conditions by activating the CB_1_ receptor [101]. An induction of melanin synthesis was also confirmed in primary human melanocytes treated with 1 µM of the cannabinoids *R*(+)-methanandamide, AEA, ACEA and 2-AG, while the CB_2_ agonist JWH133 did not increase melanogenesis [89]. Using siRNA transfections targeting CB_1_ and receptor antagonists, the latter work demonstrated that *R*(+)-methanandamide triggers melanogenesis via CB_1_ receptor activation and a p38- and p42/44-MAPK-mediated pathway involving tyrosinase expression via the master regulator MITF. In addition, a recent study examined the effects of THC and CBD on human epidermal melanocytes derived from light and dark pigmented cells. In light pigmented cells, both cannabinoids increased melanin synthesis and dendriticity, as evidenced by an increase in total dendrite length [102], a surrogate measure of melanosome secretion as a step in the melanogenesis cycle. However, in dark pigmented cells, none of the cannabinoids significantly altered dendriticity, although they retained their melanin synthesis-inducing effect, which in the case of THC was faintly inhibited by the CB_1_ receptor antagonist SR141716. Interestingly, the authors found an increased production of reactive oxygen species (ROS) by both cannabinoids in light pigmented cells, which was not observed in dark pigmented cells. These findings suggest that individuals with dark pigmented melanocytes may be protected from UV light-induced oxidative stress after treatment with THC and CBD, whereas in individuals with light pigmented skin, increased skin pigmentation is associated with higher oxidative stress when exposed to THC or CBD. 

#### 3.2.2. Influence on Wound Healing 

An essential endogenous process in which the endocannabinoid system appears to play an important role is skin wound healing. In this context, numerous preclinical studies confirm the effects of cannabinoid receptor modulation on the signaling pathways responsible for re-epithelialization and scar formation. Thus, it can be assumed that the endocannabinoid system is an integral part of this physiological process. The involvement of the endocannabinoid system in wound healing processes accordingly forms the basis for the use of cannabinoids in wound care, as recently outlined [103].

##### Cannabinoid Receptor Knockout Models

The importance of the role of cannabinoid receptors is particularly evident in the knockout model. During early wound healing, CB_1_ knockout mice exhibit an impaired wound closure response associated with increased levels of interleukin (IL)-6, tumour necrosis factor (TNF)-α and monocyte chemoattractant protein (MCP)-1/CC-chemokine ligand (CCL)2 [104]. In the latter investigation, knockout of CB_2_ was found to increase IL-6 and TNF-α without affecting tissue regeneration. 

##### Role of CB_2_ Receptor Activation—Results from In Vivo Experiments

A study using a mouse skin incision wound model found that activation of the CB_2_ receptor by the selective CB_2_ receptor agonists GP1a (1-(2,4-dichlorophenyl)-6-methyl-N-piperidin-1-yl-4H-indeno [1,2-c]pyrazole-3-carboxamide) and JWH-133 (Figure 1) reduces the infiltration of M1 macrophages and increases the infiltration of M2 macrophages during skin wound healing, thereby reducing skin inflammation [105]. In this context, a separate investigation by the same group observed accelerated re-epithelialisation after treatment of mice with GP1a [106]. In the latter study, the authors described that activation of the CB_2_ receptor reduced neutrophil and macrophage infiltration and increased the expression of MCP-1/CCL2, stromal cell-derived factor (SDF)-1, IL-6, IL-1β, TNF-α, transforming growth factor (TGF)-β1 and vascular endothelial growth factor (VEGF)-A, but increased keratinocyte proliferation and migration, ultimately leading to accelerated re-epithelialisation of wounds and reduced scar formation [106]. In a model using BALB/c mice, GP1a was used again to reduce the number of collagen I-positive fibroblast cells, resulting in a substantial decrease in skin thickness in the wounded areas. During skin wound healing, levels of TGF-β1 and TGF-β receptor I (TβRI) were decreased by GP1a and increased by the CB_2_ receptor antagonist AM-630, suggesting that TGF-β signalling is involved in CB_2_ receptor action. As downstream mediators of the canonical TGF-β signalling pathway, phosphorylation of small mothers against decapentaplegic homolog (Smad) 3 was downregulated by GP1a and increased by AM-630, while Smad7 was increased by GP1a in skin samples [107]. Another in vivo study using a mouse model of skin excision wounds showed that a GP1a-containing gel using triglycerol monostearate hydrogel prepared by a specific method [108] was able to reduce inflammation and fibrogenesis and to promote wound enclosure and re-epithelialisation [109]. In addition, the CB_2_ receptor agonist β-caryophyllene was found to enhance re-epithelialisation in a mouse model of cut wound repair due to increased cell proliferation and cell migration from intact skin near the wound towards the wound centre [110]. Interestingly, this observation was only made in female mice, while β-caryophyllene did not result in faster wound closure in male mice. When β-caryophyllene was combined with the CB_2_ receptor antagonist AM-630, the effect was partially but not significantly reduced. However, in this study, the CB_2_ agonist JWH133 also significantly promoted re-epithelialisation compared to the controls. When considered as a whole, CB_2_ receptor activation appears to be an effective strategy to achieve wound healing effects.

##### Effects of ACEA and Adelmidrol

Interestingly, there is little research specifically describing the effects of CB_1_ receptor activation on wound healing. However, the enhancement of wound healing by CB_1_ activation is supported by a study reporting that activation of the CB_1_ receptor by ACEA reduced the levels of keratin 6 and 16 in an organ culture of human skin [111]. The latter keratins are functionally important proteins for the regulation of epithelial wound healing, but on the other hand are upregulated in psoriasis [111]. In addition, the PEA-like cannabimimetic adelmidrol (Figure 1) was reported to stimulate skin wound healing in a diabetic mouse model [112]. However, this study did not investigate which receptors mediate this effect. Since adelmidrol has been described as a PPARγ agonist [113], it seems likely that this receptor is involved.

##### Role of FAAH—Results from In Vivo Experiments

A comprehensive study on the involvement of FAAH in wound healing processes demonstrated that genetic or pharmacological disruption of FAAH activity accelerates skin wound healing in vivo and stimulates human keratinocyte motility [114]. Accordingly, healing of excision wounds was accelerated in mice lacking the FAAH gene compared to wild-type control mice. However, the authors of the latter study reported that the mechanism of action was related to the epidermal growth factor receptor (EGFR) and an increase in intracellular calcium levels in response to TRPV1 rather than to the modulation of cannabinoid receptors. The authors of the latter study also identified the amides of long-chain fatty acids with taurine (*N*-acyl taurines) as substrates of FAAH and mediators of the observed accelerated wound healing.

##### Role of the Endocannabinoid System in Cell Migration—Results from In Vitro Experiments

Numerous investigations conducted with skin-derived cells have provided more detailed insight into the mechanisms of wound healing by cannabinoids. In one study, THC was reported to induce migration of human primary mesenchymal stem cells, which served as in vitro system to demonstrate the regenerative effects of cannabinoids, via CB_1_ receptor-dependent activation of p42/44 MAPK [115]. A similar effect on stem cells has been observed for CBD, but mediated via activation of the CB_2_ receptor and antagonisation of GPR55, leading to downstream activation of p42/44 MAPK, which ultimately increased migratory capacity [116]. Furthermore, using the same cells, it was reported that inhibition of FAAH by URB597 and *N*-arachidonoyl serotonin (AA-5HT) also led to increased stem cell migration via activation of p42/44 MAPK, which in turn resulted in downstream activation of PPARα [117]. One caveat, however, is that the cells used in these studies were not derived from skin tissue but from underlying adipose tissue. Another study reported that a hydrophobic extract of flax fibres containing CBD activates skin cell matrix remodelling and promotes fibroblast and keratinocyte migration as crucial parameters of wound healing [118]. Regarding the effect of cannabinoids on cell migration, primary cultured fibroblasts and keratinocytes from wild-type C57BL/6 mice exposed to β-caryophyllene showed higher chemotactic responses, while β-caryophyllene displayed no such effects in cells from CB_2_ knockout mice [110].

Interestingly, the enhanced wound healing by cannabinoids in skin wounds and the associated cannabinoid-induced migration of human keratinocytes and stem cells seem to be in contrast to their effect on malignant skin cells. For example, in melanoma cells, the CB_2_ agonist JWH-133 has been shown to lead to inhibition of transendothelial migration [119]. In another work, a standardised cannabis extract also exhibited a migration-inhibiting effect on melanoma cells [120]. The differences in this regard could be due to the fact that, for example, the CB_2_ receptor is upregulated in melanoma tissue [121] and therefore corresponding agonists achieve greater efficacy in malignant tissue in some studies. Moreover, cannabinoid compounds often exert different effects at higher concentrations than at lower ones. For the endocannabinoid-like substance OEA, for example, it could be shown that above a concentration of 2 µM, the migration of melanoma cells is inhibited, while below this concentration, migration is increased [122]. On the other hand, a recent study found that CBD and THC at a concentration of 5 µM each did not significantly alter the migration of A375 melanoma cells [123], suggesting that the effects on skin cancer motility depend on the cannabinoid used. Considering that genetic deletion of the CB_1_ receptor resulted in reduced rather than increased migration of melanoma cell lines [124], it is tempting to speculate that the cannabinoid receptor-mediated effect on the migration of normal physiological skin cells is in fact no different from the effect on the migration of skin cancer migration.

##### On the Path to Clinical Use

The finding that cannabinoids offer preclinical benefits in models of scarring in wound healing processes as well as in diabetic wound healing suggests that cannabinoids could be used in cosmetic support therapy for scar-free wound healing as well as in the critical treatment of diabetic wounds. More and more promising new formulations are being developed. Thus, a CBD-containing hydrogel dressing based on the ion-crosslinked interaction between Zn^2+^ ions and the alginate polymer was recently reported to accelerate wound healing in vivo [125]. Based on the promising preclinical results, corresponding cannabinoid effects have meanwhile been investigated in patients with venous leg ulcers [126]. Here, liposomal formulations with a mixture of CBD, THC and other substances proved to be beneficial. Details can be found below in chapter 5.

#### 3.2.3. Influence on Cutaneous Barrier Function

Recent studies have also shown that cannabinoid receptors are involved in important barrier functions, the dysregulation of which is in turn a crucial factor in the pathophysiology of diseases such as psoriasis and acne. Accordingly, CB_1_ receptor knockout mice were found to exhibit delayed permeability barrier recovery compared to wild-type mice, while CB_2_ receptor knockout mice had accelerated barrier recovery [127]. Consistent with these findings on the role of the CB_1_ receptor, a recent study reported that acute barrier disruption induced by repeated tape stripping and acute inflammation induced by TPA were inhibited by the newly synthesised CB_1_ agonist α-oleoyl-oleoylamine serine (α-OOS) [128]. Another study has also adressed the role of endocannabinoid reuptake and degradation on skin barrier function and skin inflammation in vivo [129]. Here, WOL067-531, an inhibitor of endocannabinoid reuptake with no appreciable effect on FAAH activity, inhibited barrier repair functions [129]. Methodologically, the latter study was designed so that barrier disruption was likewise achieved by tape stripping and skin inflammation was achieved by a patch test with sodium dodecyl sulphate, which is used as a positive reference control in such experiments [130]. In the experiments on skin inflammation, a reduction in epidermal proliferation and the number of proinflammatory cells as well as a reduction in β-defensins by WOL067-531 and additionally by the selective FAAH inhibitor, WOBE440, was observed [129]. β-Defensins are an important component of the antimicrobial barrier and defence [131].

A positive influence of cannabinoid compounds has also been observed where inflammatory stimuli disrupt epidermal permeability barrier function, as demonstrated for *N*-palmitoyl serinol (Figure 1), a derivative of 2-palmitoyl glycerol [132]. Here, *N*-palmitoyl serinol increased total ceramide production in human epidermal keratinocytes, which are a critical structural component of the epidermal permeability barrier, in a CB_1_ receptor-dependent manner [132]. Results beyond skin cell analysis have confirmed the effect of cannabinoids on cellular ceramide production [133,134]. In a recent study, CBD was also found to increase the expression of aquaporin 3 (AQP3) in the skin of mice, which plays an important role in water retention in the skin and is therefore likely responsible for the moisturising effect of CBD [135]. Here, CBD-induced AQP3 expression was associated with increased skin water content without any change in transepidermal water loss, indicating the maintenance of normal barrier function. However, this study did not investigate the extent to which cannabinoid-triggered receptors contribute to this effect.

Since cannabinoids have a wide range of interactions with PPARs, as described above, the results of the studies on the role of PPARs on barrier function are also relevant. In this context, an investigation revealed that PPARγ knockout mice exhibit a disruption of the permeability barrier [136]. The latter is associated with gene regulations related to lipid metabolism and cutaneous lipid barrier function, such as the downregulation of enzymes of the very long chain fatty acid group (Elovl 3, 5 and 6), which are important factors of the lipid permeability barrier of the stratum corneum [137,138]. In addition, PPARα was found to protect epidermal barrier function (for review see [139]). However, although there are many studies on PPARγ- and PPARα agonists that do not belong to the cannabinoid group, it is not yet clear to what extent cannabinoids influence the barrier function of the skin via this receptor. Furthermore, based on current data, it is difficult to clearly determine which receptor plays the most important role in cannabinoid-mediated homeostasis of skin barrier function. However, it is tempting to speculate that cannabinoids with a network of different receptors may favour the stability of skin function in certain circumstances.

#### 3.2.4. Influence on Sebocyte Biology

Cannabinoids may also play a crucial role in the function of the sebaceous glands, which have an intense lipid metabolism. The main product of the sebaceous gland, sebum, is an oily substance enriched with lipids, mainly consisting of triglycerides, free fatty acids, wax esters, cholesterol and squalene, which protects the skin (for review see [138]). In this context, endocannabinoids were found to upregulate the expression of key genes involved in lipid synthesis via CB_2_ in the human SZ95 sebocyte cell line, which does not express CB_1_ receptors [88]. In addition, inhibition of endocannabinoid uptake has been shown to have anti-inflammatory effects and to increase sebum production [140]. Among further cannabinoid receptor targets, PPARs are classical lipid regulators in sebocytes, as studied by others (for review see [141]). PPARα and PPARγ were found to stimulate sebum production [142], stimulate sebocyte proliferation and inhibit terminal differentiation and apoptosis, thereby preventing the release of acne-associated lipids [143], a property that serves as a rationale for activation of these PPARs in the treatment of acne. Consistent with this notion, PPARγ knockout mice even exhibited complete absence of sebaceous glands [144]. In contrast to the signalling pathways leading to the potentially beneficial effects described above, the TRPV3 receptor can lead to an unfavourable reduction in sebocyte lipogenesis. Thus, activation of TRPV3 resulted in inhibition of lipogenesis and stimulated the production of the proinflammatory cytokines IL-1, IL-6, IL-8 and TNF-α in human sebaceous gland-derived SZ95 sebocytes [145]. A similar increase in proinflammatory cytokines after TRPV3 activation has also been observed in keratinocytes [146], suggesting that it is a cell type-independent general mechanism of this receptor. However, the extent to which the above-mentioned TRPV3-activating cannabinoids can limit their own anti-inflammatory effect via TRPV3 activation has not yet been investigated in skin cells.

With regard to the effect of cannabinoids on sebocyte function, a recent review even suggests that cannabinoids may act in the context of Parkinson’s disease treatment by both reducing the pathogenesis associated with Parkinson’s disease and inhibiting seborrheic dermatitis, which is a common symptom of Parkinson’s disease [147].

#### 3.2.5. Influence on Hair Follicle Biology

Yet, another function attributed to the endocannabinoid system in the skin may be the control of hair growth. Accordingly, one study reported that AEA and THC inhibit hair shaft elongation and proliferation of hair matrix keratinocytes and stimulate apoptosis of cultured human hair follicles in an organ culture system [86]. In addition, a recent investigation using microdissected hair follicles cultured in toto to preserve the intact epithelial stem cell niche demonstrated that CB_1_ receptor activation induces the proliferation of hair follicle epithelial progenitor cells ex vivo [148]. The authors also found here that intrafollicular knockout of the CB_1_ receptor or repeated treatment with a CB_1_ antagonist leads to a significant reduction in the number of human epithelial stem cells in the hair follicle and that CB_1_ knockout mice have fewer epithelial stem cells in the bulge. The study therefore concludes that tonic CB_1_ activation is necessary for the survival of human hair follicle stem cells. Considering opposing effects of CB_1_ receptor activation and inhibition, it can be concluded that CB_1_ agonists could help reduce unwanted hair growth as CB_1_ receptor activation triggers apoptosis-induced premature catagenic regression of hair follicles, while CB_1_ antagonists could be used as a therapeutic option for alopecia [86].

In recent years, the influence of PPARs on hair growth has also been increasingly studied. In this context, PPARγ knockout mice were found to lead to upregulation of genes involved in inflammation and keratinisation, with increased epidermal acanthosis, spongiosis and parakeratosis. In addition, loss of PPARγ in the epidermis has been linked to the regulation of other marker genes associated with the asebia phenotype, increased transepidermal water loss, alopecia, dandruff and inflammatory skin lesions [136]. Here, in PPARγ knockout mice, an aseptic skin phenotype with alopecia was observed, which is consistent with the results from other authors [144]. Furthermore, tissue-specific deletion of PPARγ in bulb stem cells resulted in scarring alopecia and sebaceous gland hypoplasia resembling human lichen planopilaris, a scarring (cicatricial) alopecia in which PPARγ is also downregulated [149]. The latter study also demonstrated that non-cannabinoid receptor agonists at the PPARγ, i.e., ciglitazone, rosiglitazone, pioglitazone and troglitazone, induce peroxin 16 (PEX16) gene expression in keratinocytes of the outer root sheath. PEX16 gene product deficencies were found to be associated with peroxisomes disappearance (for review see [150]) that is a marker of the lymphocytic cicatricial or scarring alopecia disease, lichen planopilaris. Therefore, PPARγ as a target of various endo- and exocannabinoids could be an important parameter for hair follicle homeostasis.

#### 3.2.6. Influence on the Photoexposed Epithelium

In an earlier review article of our group on the anticarcinogenic effects of cannabinoids on skin cancer, the preventive effects of cannabinoid compounds on sunlight-induced carcinogenesis were already discussed [1]. The basis of these effects is, as already stated above in connection with UV light-induced melanogenesis, the protective function of the endocannabinoid system in the case of physicochemically induced skin damage by UV light. However, a study on this revealed that UV irradiation was more likely to induce papillomas in wild-type mice than in CB_1_ and CB_2_ double-knockout mice, which accordingly showed a lower susceptibility to the inflammatory response triggered by UV light with a markedly attenuated activation of the transcription factor Nuclear Factor-κB (NF-κB), a decreased production of TNF-α and a far more resistant epidermis. Consequently, the results of this work suggested that cannabinoid receptors act as oncogenes in this process [151]. Nevertheless, several studies have now reached the consensus that CBD among cannabinoid compounds reduces the harmful effects of UV radiation. Accordingly, some more detailed proteomic analyses revealed a protective regulatory profile of CBD in human skin fibroblasts in this context. This was later confirmed in vivo using UV-irradiated rat skin, where CBD caused proteostasis in keratinocytes and reversed or greatly reduced regulations associated with UV light-induced inflammation [152]. In line with this notion, another study showed that CBD increased the activity of antioxidant enzymes such as superoxide dismutase and thioredoxin reductase in keratinocytes exposed to UV radiation [153]. Besides CBD, CBG was also shown to inhibit the release of proinflammatory cytokines from UV light-exposed human skin fibroblasts and normal human epidermal keratinocytes [154]. However, this study did not address the role of cannabinoid-triggered receptors in this process.

An example of a disease related to chronic UV light exposure is actinic keratosis, a premalignant intraepithelial skin lesion that develops in light-exposed skin areas and for which cannabinoids may be helpful. The expression of FAAH and TRPV1 in the actinic keratosis cell line HT-297.T has been described previously [155]. Furthermore, a study reported downregulation of CB_1_ receptor expression in 9 out of 9 samples from patients with seborrhoeic keratosis [156], a skin disease similar to actinic keratosis characterised by cellular dysfunction of mast cells [157], whose activities have been shown to be regulated by endocannabinoids [158]. However, there are no studies on the use of cannabinoid compounds in actinic keratosis. Current therapies include 5-fluorouracil, photodynamic therapy, imiquimod, chemical peeling with trichloroacetic acid and diclofenac gel [159]. Cannabinoids could usefully expand this limited pharmacological armamentarium.

#### 3.2.7. Influence on Cutaneous Pain

A further important regulatory circuit, largely determined by the endocannabinoid system, is the control of pain transmission and perception in the skin. Thus, more than two decades ago, AEA was found to reduce pain behaviour in response to chemical damage to the skin via the CB_1_ receptor, suggesting a possible use of cannabinoids in painful skin conditions [84]. A supporting factor contributing to pain relief in this study was the release of PEA, which surprisingly activated peripheral CB_2_ receptors here. Accordingly, treatment with cannabinoids may have a general benefit for patients with painful skin conditions. As shown in various case reports, e.g., in epidermolysis bullosa and pyoderma gangrenosum (see Section 4.8. and Section 4.9.), the analgesic effect of cannabinoids in particular is an essential factor that improves the patients’ quality of life.

#### 3.2.8. Influence on Keratinisation

There are a number of regulations of keratins by cannabinoids that have been demonstrated so far. In a review on the effects of neuroendocrine regulation of keratin expression on pathological conditions, keratin 6, 16 and 17 are described as mediators of psoriasis, lichen planus, pachyonychia congenita, but also as beneficial parameters in wound healing [160]. Other keratins that will play a role in the following are keratin 1 and 10, which are likewise associated with psoriasis and lichen planus, but also with epidermolytic ichthyosis [160].

What is known about the role of cannabinoid receptors in keratin regulation is that the CB_1_ agonist ACEA decreased the expression of keratin 6 and 16 in organ-cultured human skin, which was associated with reduced proliferation of human epidermal keratinocytes [111]. Here, the proliferation of keratinocytes and the downregulation of keratin 6 by ACEA was partially inhibited by a CB_1_ receptor antagonist. Topical CBD, on the other hand, tested for its suitability as a therapeutic option for keratin disorders, increased keratinocyte proliferation and keratins 16 and 17 [161]. However, this study did not investigate the cannabinoid-driven receptors that trigger these regulations. Considering these results and the fact that a disease such as psoriasis is characterised by hyperproliferation of keratinocytes, the authors of the latter study considered that the use of CBD in psoriasis should be taken with caution. Another study reported a reduction in keratin 10 by treatment of differentiating keratinocytes with CBD that was reversed by the CB_1_ antagonist SR141716 [162]. Consistent with this, AEA was found to downregulate keratin 1 and 10 in differentiating HaCaT keratinocytes via activation of the CB_1_ receptor [163]. The authors concluded that the endocannabinoid system significantly controls epidermal differentiation, with CB_1_ receptor activation inhibiting keratinocyte differentiation, which was also observed after treatment of differentiating keratinocytes with 2-AG, NADA and ACEA. These results also confirm an earlier study by the research group in which endogenous AEA levels in differentiating keratinocytes were reduced by an increase in FAAH expression, which was accompanied by reduced formation of cornified envelopes as a marker of keratinocyte differentiation [164].

#### 3.2.9. Influence on Skin Ageing Processes

Early work on the involvement of cannabinoid receptors in skin ageing already showed that CB_1_ knockout mice exhibited clear signs of earlier skin ageing with reduced width of the subdermal fat layer compared to wild-type mice [165]. Later work using knockout mice has shown that a lack of CB_1_ receptors in the skin is associated with accelerated skin ageing due to increased production of ROS, a decrease in antioxidant defenses and a pro-inflammatory environment [166]. This finding could thus provide the basis for the use of topical cannabinoid applications to reduce skin ageing. In the cited study, collagen levels in the skin of CB_1_ knockout mice were decreased, suggesting that the CB_1_ receptor may be an endogenous “anti-ageing receptor”. However, it should be noted that these are animal experiments on a diabetes model, the transferability of which to humans would still have to be proven.

A recent study has further demonstrated that the CB_1_ receptor on the outer mitochondrial membrane in human epidermal keratinocytes is a negative regulator of mitochondrial activity in the human epidermis, the activation of which could reduce excessive mitochondrial ROS production and thus exert a protective function against skin ageing or photodestruction [167]. In addition, extracts derived from *Cannabis sativa* have recently been shown to have an antioxidant capacity associated with an increase in superoxide dismutase, free radical scavenging, reduced elastase and collagenase activity, as well as in vivo water binding and a long-lasting moisturising effect [168]. However, this study did not address the role of cannabinoid receptors, and for the moisturising effect, the authors assume that cannabinoids act like an occlusive film on the skin surface and retain water.

### 3.3. Regulation of the Endocannabinoid System in Skin Diseases

#### 3.3.1. Endocannabinoids and Classic Cannabinoid Receptors

The role of the elements of the endocannabinoid system becomes even clearer when one considers how dynamically endocannabinoids and cannabinoid receptors are regulated in the context of skin diseases. A recent study revealed that AEA and 2-AG were elevated 2- to 4-fold in fibrotic mouse skin compared to control skin [169]. Induction of 2-AG has also been demonstrated to be detectable following 12-O-tetradecanoylphorbol-13-acetate (TPA)-induced ear inflammation of mice [170] and in a mouse model of contact dermatitis [171]. In another report, higher concentrations of AEA and 2-AG were also found in the plasma of patients with psoriasis vulgaris and psoriatic arthritis [172]. In addition, AEA and 2-AG have been reported to be reduced in keratinocytes from psoriasis patients, while PEA levels are increased [7]. Finally, in the model for 1-fluoro-2,4-dinitrobenzene (DNFB)-induced contact dermatitis, AEA and 2-AG were both shown to be upregulated in exposed ear samples [3]. Upregulation of 2-AG has also been demonstrated in skin lesions of mite antigen-induced atopic dermatitis in mice [5].

Upregulation of the CB_1_ receptor was detected in granulocytes of psoriatic arthritis patients, while the CB_2_ receptor appeared to be upregulated only in granulocytes of psoriasis vulgaris patients [172]. In skin biopsies from psoriasis and atopic dermatitis patients, another recent comprehensive investigation focusing on mRNA profiles of itchy lesional skin found both cannabinoid receptors downregulated here [173]. Interestingly, there is a veterinary study on this topic that has shown that both cannabinoid receptors are upregulated in the skin of dogs with atopic dermatitis compared to the skin of healthy dogs [174]. However, no quantitative assessment was performed in this study. The focus here was on the distribution of cannabinoid receptors in the individual components of the skin, with strong CB_1_ immunoreactivity observed in the keratinocytes of the suprabasal spinous layers of the epidermis and strong CB_2_ immunoreactivity in the basal, suprabasal and granular layers of the epidermis in dogs with atopic dermatitis. In addition, it was observed that CB_1_ receptor immunoreactivity was significantly downregulated in human epidermis at sites of seborrhoeic keratosis compared to the marginal lesion, leading to a subsequent upregulation of stem cell factor (SCF), which activated mast cells and increased the severity of seborrhoeic keratosis. In this context, endocannabinoids have been defined as important neuroendocrine regulators for the maintenance of skin homeostasis [156]. In a recent report, CB_2_ receptor expression was observed to be increased in dermatomyositis in certain cell types found in the skin, such as dendritic cells, B-cells, T-cells and macrophages, which produce IL-31, IL-4, interferon (IFN)-γ and IFN-β, compared to healthy skin [8]. Another investigation showed that cannabinoid CB_2_ receptors are upregulated in neutrophils, macrophages and myofibroblasts in a time-dependent manner during skin wound healing in mice [175]. Moreover, a recent study using primary cultures of adult human fibroblasts revealed that TGF-β, a profibrotic mediator, significantly increases the expression of CB_1_ [176] and CB_2_ receptors [177], suggesting that both receptors may play a role in fibrotic diseases of the skin and thus represent potential targets for pharmacotherapeutic interventions. Moreover, in mice in the above-mentioned model of streptozotocin-induced type 1 diabetes associated with accelerated skin ageing, the CB_1_ receptor has been shown to be downregulated, which was accompanied by higher expression of pro-inflammatory markers [166]. In the in vivo model of DNFB-induced contact dermatitis, the CB_1_ receptor was further found to be downregulated and the CB_2_ receptor upregulated in the affected ear tissues [3]. Finally, for the CB_2_ receptor, upregulation was detected in psoriatic skin lesions in a mouse model of imiquimod-induced psoriasis [178].

In general, it is difficult to assess whether the regulations of these receptors are causally related to the disease or represent epiphenomena or are counter-regulations to maintain homeostasis. The known regulations of endocannabinoids and cannabinioid receptors in the context of skin diseases can only ever hint at functional tendencies. In order to derive more precise functional implications, it would actually first be necessary to qualitatively and quantitatively assign the regulations in the respective disease context to the individual components and cell types of the skin through immunohistochemical analyses of skin sections. In any case, the current knowledge about the regulations already illustrates the close involvement of these receptors in physiological and pathophysiological processes.

Table 1 summarises selected regulations of elements of the classic endocannabinoid system associated with pathogenic changes of the skin.

#### 3.3.2. Extended Endocannabinoid System

Considering the role of cannabinoid targets beyond the classic cannabinoid receptors CB_1_ and CB_2_, such as TRP channels, PPARs, GPR55, GPR119 and FAAH, there are numerous other proven or suspected mechanisms of cannabinoid action in skin diseases. There are also multiple findings on how these targets are regulated in skin diseases.

Table 2 provides an overview of the regulation of these cannabinoid-modulated receptors of the extended endocannabinoid system under the indicated pathophysiological alterations.

##### TRP Channels

The regulations of the individual TRP channels appear to be complex and differentiated, with some upregulated and others downregulated in a pathophysiological context. Analysis of skin biopsies revealed that TRPV2 was downregulated in itchy skin regions of psoriasis patients, while FAAH1, TRPV1 and TRPV3 were upregulated. In contrast, in skin biopsies from patients with atopic dermatitis, TRPV1 and TRPV2 were upregulated and TRPV3 and TRPM8 were downregulated [173]. Immunohistochemical analyses confirmed these data for selected regulations, including TRPV1, which is upregulated in both atopic dermatitis and psoriasis biopsies, suggesting that this cannabinoid target may represent an underappreciated mediator of itch as a parameter of a common “itch-scriptome” match [173]. In this context, TRPV1 was also found to contribute to adverse pain-mediating effects in an in vitro model of psoralen UVA (PUVA) therapy used to treat vitiligo, psoriasis, eczema and mycosis fungoides [189].

Based on studies indicating interaction of various phytocannabinoids with TRP channels [58], the authors of a recent publication hypothesised that phytocannabinoids could be a therapeutic option for the treatment of rosacea, in which such ion channels play an important role in pathogenesis [190]. Consistent with this notion, TRPV channels are dynamically regulated in different subtypes of rosacea. Accordingly, TRPV1, 2 and 3 appear to be upregulated in erythematotelangiectatic rosacea and TRPV2 and 3 in papulopustular rosacea [179]. In phymatous rosacea, on the other hand, TRPV1, 3 and 4 are upregulated, while TRPV2 is downregulated [179]. In line with the upregulation of TRPV1 in dysregulated skin, TRPV1 antagonists have been reported to reduce itching behaviours [191] and improve barrier repair [191,192]. In summary, although TRPV2 was downregulated in pruritic skin regions of psoriasis and phymatous rosacea and TRPV3 was downregulated in skin biopsies from patients with atopic dermatitis, TRP channels appear to be upregulated in most skin disease studies. Therefore, it is reasonable to assume that TRP channels may play a role in symptoms, especially pain. In view of this, it cannot be ruled out that cannabinoids that act agonistically on TRPV1, such as CBD, may partially attenuate their beneficial effects elicited by other receptors.

TRPV4 has been found to mediate dry-skin-induced pruritis [193] and chronic pruritis [180]. Accordingly, in the latter investigation, TRPV4 expression was found to be significantly increased in skin biopsies from patients with chronic idiopathic pruritus compared to skin from healthy control subjects.

Finally, a possible involvement of TRPV3 downregulation has been suggested in patients with middle ear cholesteatoma. Middle ear cholesteatoma is a chronic purulent inflammation of the middle ear with bone destruction as another disease involving epidermal cells, which in most cases results from the ingrowth of squamous epithelium from the external auditory canal into the middle ear [194].

##### GPR55, GPR119 and GPR18

A recent study reported that higher levels of GPR55 are found in granulocytes from psoriasis and psoriatic arthritis patients than in the control group [172], suggesting that cannabinoids with GPR55-antagonising effects, as is the case with CBD [61], may be beneficial for these indications. In addition, GPR119, which can be activated by OEA, has been found to be downregulated in the sebaceous glands of acne patients [99]. In the latter study, the OEA/GPR119 pathway was described as an essential mechanism of seborrhoea and acne, with OEA causing a lipogenic effect, increasing cellular granulation and switching sebocytes to a proinflammatory phenotype, suggesting pharmacological blockade of GPR119 as a potential treatment option [99]. However, the fact that prolipogenic GPR119 is downregulated in the sebaceous glands of acne patients seems to contradict the acne-promoting effect of GPR119. Here, the authors suspect an over-activation of the receptor in the early and not in the late stages of acne or a downregulation due to secondary compensatory mechanisms in response to excessive acneogenic sebum production.

Regarding the effect of cannabinoids at GPR18 in skin diseases, there are currently no data on a possible pharmacological effect, although both GPR18 [195] and the endogenously synthesised ligand *N*-palmitoyl glycine [67] have been detected in skin tissue.

##### PPARs

An mRNA profiling analysis of skin biopsies from patients with atopic dermatitis and psoriasis revealed that PPARα and PPARγ are downregulated in the itchy skin regions of psoriasis patients, while PPARα remains unchanged in patients with atopic dermatitis and PPARγ increases by 24% [173]. Downregulation of both PPAR subtypes was confirmed in skin biopsies from patients with systemic sclerosis [184]. Another study confirmed the downregulation of PPARγ in explanted fibroblasts of lesional skin from patients with systemic sclerosis [98]. In addition, PPARγ has been reported to be downregulated in UV-irradiated damaged skin [185], in systemic lupus erythematosus patients with skin lesions [187] and in psoriatic and atopic lesions [186]. An investigation of 100 acne patients compared to 100 healthy subjects further found that the Pro12Ala polymorphism of the PPARγ gene, which results in decreased PPARγ transcriptional activity, is associated with a lower risk of acne vulgaris [196]. In line with the importance of PPARγ in atopic diseases, another study suggested PPARγ activation as a potential therapeutic option for the treatment of allergic rhinitis [197].

With respect to PPARα, its expression has been found to be reduced in eczematous skin from patients with atopic dermatitis compared to skin from non-atopic donors [182]. Likewise, PPARα has been shown to be downregulated in affected skin regions in melasma [183] and in human skin lesion with actinic keratosis compared to healthy skin [181]. Interesting new aspects could also result from veterinary findings. For example, it has been shown that in cats with eosinophilic dermatitis, PPARα accumulates in the basal and upper cells of the hyperplasmatic epidermis and in keratinocytes adjacent to the ulcer in affected skin compared to healthy controls [92].

Finally, one study reported that PPARδ appears upregulated in the lesions of patients with psoriasis [188] which may therefore serve as another possible target effected by cannabinoids.

## 4. Selected Skin Diseases—Pharmacotherapy and Effect of Cannabinoids

Several data on cannabinoid effects have now been collected using in vitro and in vivo models of skin diseases such as androgenetic alopecia, atopic dermatitis, allergic contact dermatitis, psoriasis, acne, systemic sclerosis, dermatomyositis, epidermolysis bullosa and pyoderma gangrenosum, acute inflammation, post-herpetic neuralgia and various keratin diseases. Since the diseases discussed below for which cannabinoids have been tested almost all have inflammation as a common feature, each of which has a different significance for the development and progression of the corresponding diseases, some historical background on the anti-inflammatory effects of cannabinoids will be presented first. Since pruritus is symptomatic in most of these diseases, the following remarks on the pharmacotherapeutic possibilities of cannabinoids in the specific diseases are also preceded by a short section on the general antipruritic effect of cannabinoids.

A Brief Historical Overview on Anti-Inflammatory Effects of Cannabinoids

Consistent with this exposed homeostatic function of the endocannabinoid system in the skin, numerous publications have described a potential therapeutic value of cannabinoids in the treatment of inflammatory skin diseases. An important part of the anti-inflammatory action of cannabinoids is their effect on various cells of the immune system, which was summarised in a recently published review [198].

The first descriptions of the use of cannabis may date back to ancient Egyptian sources, where cannabis was probably used as an anti-inflammatory option to treat skin swelling [199]. Well documented in ancient writings is the use of cannabis for burns by Gaius Plinius Secundus (AD 23/24–79) [200]. The use of cannabis for burns appears again and again over the centuries, for example in 1698 with Nicholas Lémery, who recommended hemp for the treatment of burns [201]. Although the analgesic effect of cannabinoids in burns is still a subject of scientific discussion today [202], the use of cannabinoid compounds for this indication has rather faded into the background.

Insights into mechanisms of anti-inflammatory effects of cannabinoids were gained long before the discovery of cannabinoid receptors. Thus, experiments from the early 1970s already demonstrated an anti-oedematous and anti-arthritic effect of THC [203]. Later experiments testing the anti-oedema effect in rats showed that the inhibitory effect of THC was limited to oedema induced by carrageenan, kaolin and sodium urate, while hydrocortisone and aspirin were effective against a wider range of oedemas, including those induced by histamine and bradykinin. This suggests a different antiphlogistic mode of action of THC compared to steroidal and non-steroidal anti-inflammatory drugs [204]. The inhibitory effect of cannabinoids on inflammatory pain has also been demonstrated in several other reports [205,206,207].

Interestingly, the earliest published comprehensive data on anti-inflammatory and anti-allergic effects of lipids belonging to the endocannabinoid system mainly refer to the endocannabinoid-like substance PEA and date back to the 1950s [208]. These findings were later substantiated in animal experiments, which showed that PEA reduces oedema [209,210] and inhibits mast cell activation [211]. Other results of the latter study were a PEA-induced reduction in extravasation caused by passive cutaneous anaphylaxis and a reduction in oedema in various animal models. Only later was it recognised that PEA exerts its anti-inflammatory effect via activation of PPARγ [72]. A clinical study was able to prove the benefit of PEA in temporomandibular joint inflammatory pain [212]. As a result of this and other studies, a recent meta-analysis concluded that PEA may be a useful treatment option for pain, including inflammatory pain [213]. The effect of PEA on the skin, which has now been tested in several clinical studies on skin diseases, will be discussed later.

A number of publications prove that the activation of the CB_2_ receptor causes an inhibition of the immune response. For the CB_2_ receptor agonist O-1966, a mechanism was described that could cause such an effect via the increase of the two potent anti-inflammatory parameters IL-10 and regulatory T cells (Treg) [214]. The anti-inflammatory effect of CB_2_ agonists could also be confirmed in other studies [215,216]. An atypical cannabinoid that has been shown to reduce inflammatory processes is the CB_2_ receptor agonist lenabasum, which reduces the release of IL-1β from peripheral blood mononuclear cells (PBMCs) in vitro [217] and causes a reduction in inflammatory pain in a rat model of neuropathic and inflammatory pain [218]. In terms of mechanisms of action, lenabasum was found to inhibit IL-8 promoter activity in a PPARγ-dependent manner [79], confirming the antifibrotic and anti-inflammatory effects of lenabasum via PPARγ shown by other authors [219]. In contrast, another publication investigating the inhibition of IL-6 production by lenabasum in human monocyte-derived macrophages as a potential strategy for the treatment of patients with rheumatoid arthritis and systemic lupus erythematosus reported that this effect occurs in a PPARγ-independent manner [220]. Some studies have reported that lenabasum not only blocks inflammatory processes, but also accelerates the resolution of inflammation when it has already occurred. As a novel anti-inflammatory and proresolving drug, lenabasum is currently under clinical investigation for several diseases [221,222]. One mechanism of action of lenabasum is to support the release of free arachidonic acid after activation of the CB_2_ receptor and phospholipase A_2_. As a result, higher concentrations of 15-deoxy-Δ^12,14^-prostaglandin (PG) J_2_ are formed via cyclooxygenase-2 (COX-2), causing apoptosis and the resolution of chronic inflammation via eleavated caspase-3 activity. Secondly, a metabolic pathway mediated by lipoxygenase is also thought to mediate lenabasum-triggered production of anti-inflammatory and proresolving lipoxin A_4_ and other proresolving mediators [222].

There are also studies that have investigated the involvement of the CB_1_ receptor in pro-inflammatory effects. Thus, using microdissected human scalp hair follicles in the anagen VI stage of the hair cycle, a comprehensive study reported that inhibition of CB_1_ receptors with AM-251 induces degranulation of mast cells in the connective tissue sheath, which was inhibited by AEA and ACEA [158]. Further results from this work showed that inhibition or silencing of the CB_1_ receptor stimulated the maturation of mast cells from resident progenitor cells, which was associated with an upregulation of SCF.

Moreover, THC, AEA and NAGly have been found to act as full agonists at GPR18 [68], a receptor that has been detected in naïve endothelium as well as in infiltrating leukocytes of inflamed skin [195] and has furthermore been described as receptor of the resolvin D_2_ [223], a potent immunoresolvent. However, so far it has not been investigated to what extent cannabinoids can cause an inflammation-resolving effects in the skin via a direct activation of GRP18.

A Brief Overview on Anti-Pruritic Effects of Cannabinoids

Already almost two decades ago, a case report was published on three patients suffering from treatment-resistant cholestatic related pruritus, in which treatment with THC resulted in an antipruritic effect of about 4 to 6 h [23]. In the meantime, a number of well-documented effects of cannabinoids and their derivatives on skin diseases associated with itching have been described in the literature. The importance of the endocannabinoid system in the development of pruritus was shown in a study using pharmacological and genetic approaches in a murine pruritus model. Here, subcutaneous administration of the mast cell degranulator compound 48/80 triggered an intense, concentration-dependent scratch reaction, which was reduced by THC in a CB_1_ receptor-dependent manner [224]. Furthermore, genetic deletion of FAAH in this study resulted in a reduction in scratching behaviour. This reduction in scratching behaviour was mediated independently of hypomotility. In another study, WIN 55,212-2 was shown to have an antipruritic effect by reducing serotonin-induced scratching behaviour in mice [225]. An antipruritic effect was also found for S-777469 (Figure 1), a selective CB_2_ receptor agonist containing a 3-carbamoyl-2-pyridone system with carboxylic acid at the 3-position [226]. In animal experiments, itching in mice was induced by histamine or substance P and suppressed by S-777469, which was reversed by a CB_2_ receptor antagonist [227]. Interestingly, some studies have also tested options that indirectly target the endocannabinoid system rather than acting directly on cannabinoid receptors. Accordingly, in an in vivo study, a significant decrease in both groin and overall itch was found with a reduction in skin symptom severity following allergen challenge in atopic beagles after treatment with WOL067-531, a topical endocannabinoid membrane transporter inhibitor [228].

When considering the extended endocannabinoid system, it is striking that TRP channels such as TRPV1 and TRPA1 play an important role in itch signal transduction, as highlighted in a systematic review on this topic [229]. In addition, scratching behaviour in response to glucosylsphingosine was decreased in TRPV4 knockout mice [230], and GSK1016790A, a TRPV4 channel-specific agonist, induced acute itch in mice [231]. For this reason, one can speculate whether cannabinoids also counteract their antipruritic effect via their TRP-activating properties. This would be the case with the TRPV4-activating or -sensitising cannabinoids CBDV, THCV, CBGA, CBGV, CBN and CBG [58], as well as with a higher concentration of CBD (50 µM), which also activates TRPV4 [232], and with TRPV1-activating cannabinoids such as AEA [52], CBD [53], 2-AGE [38,54], NADA [37] as well as CBG, CBC, THCV and CBGA [55]. However, as far as we know, there are no studies yet that have investigated the role of these TRP channels in the effect of cannabinoids on skin itch.

In the following chapters, the antipruritic effect of cannabinoids in numerous diseases such as allergic contact dermatitis, atopic dermatitis, psoriasis and neurogenic flare reactions will be addressed.

### 4.1. Androgenetic Alopecia

#### 4.1.1. Current Therapies of Androgenetic Alopecia

Androgenetic alopecia is a hereditary thinning of the hair with a polygenic pattern of inheritance [233], which in susceptible individuals is triggered dominantly by androgens [234,235]. Approximately half of the population develop this feature before the age of 50 [236]. Currently, there are three common drugs used to treat androgenetic alopecia, namely minoxidil and the two 5α-reductase inhibitors finasteride and dutasteride, which, according to a recent meta-analysis, are roughly equivalent in terms of average change in hair number after treatment [237]. However, the optimal treatment of androgenetic alopecia is still a matter of debate due to variable adverse effects and relatively unstable results [238]. Another option for the treatment of androgenetic alopecia is a minimum triple injection of platelet-rich plasma, which was considered a safe and effective alternative procedure for the treatment of hair loss instead of minoxidil and finasteride in a recent systematic review [239]. Although androgenetic alopecia is primarily related to androgen metabolism, the results of some studies suggest microinflammation with perifollicular lymphocytic infiltration associated with this condition [240,241], in which case the anti-inflammatory effects of cannabinoids would also come into play as a mechanism of action.

#### 4.1.2. Preclinical Findings on the Effect of Cannabinoids in Androgenetic Alopecia

Among the cannabinoid compounds, CBD has been shown to enhance hair growth (for review see [242]). As a possible mechanism of action, a recent investigation reported that CBD restores testosterone-induced loss of β-catenin in human dermal papilla cells, which is downregulated in alopecia by ubiquitination and subsequent proteasomal degradation [243]. However, the latter study did not address the involvement of CBD-triggered receptors. For CBD, which also has a negative allosteric effect on the CB_1_ receptor [29] and should therefore actually promote hair growth via this mechanism, a complex mechanism besides the modulation of the cannabinoid receptors has been demonstrated: this cannabinoid inhibits hair shaft development at a concentration of 50 µM via TRPV4 agonism, but enhances it at submicromolar concentrations via activation of the adenosine receptor [232]. Interestingly, in a study investigating the effect of newly synthesised thienyl-substituted pyrazole carboxamide derivatives as CB_1_ receptor antagonists against obesity, it was found that a compound (“compound 3”) structurally mimicking rimonabant induced exceptionally strong hair growth in C57BL/6J mice [244], supporting the assumption that CB_1_ receptor antagonists increase hair growth accordingly [86].

### 4.2. Atopic Dermatitis

#### 4.2.1. Current Therapies of Atopic Dermatitis

Atopic dermatitis is the most prevalent inflammatory skin disease in developed countries, with symptoms including skin redness, peeling, lichenification and pruritus that interfere with daily life and especially sleep [245]. Characteristic of atopic dermatitis is hypertrophy of sensory nerve fibres [246] and release of calcitonin gene-related peptide (CGRP) as a critical mediator of itch and flare-ups occurring in infiltrating inflammatory cells in the late phase of the skin reaction [247]. In addition, histamine receptor 4 is upregulated on CD4-positive T cells from patients with atopic dermatitis, which is associated with the induction of IL-31, a major mediator of itch [248].

Symptom treatment of mild forms of atopic dermatitis is currently carried out with emollients and low potency topical steroids, while severe types are treated with oral corticosteroids, cyclosporine (for review see [249]) and other immunosuppressants such as azathioprine [250], methotrexate [251,252] and the calcineurin inhibitors tacrolimus and pimecrolimus, which were approved as topical treatment options for atopic dermatitis about two decades ago [253]. An important biologic for the treatment of atopic dermatitis is dupilumab, an IL-4 receptor-α inhibitor that blocks both IL-4 and IL-13 activity and is approved for the treatment of moderate-to-severe atopic dermatitis in patients 6 years of age and older (for review see [254]). Other biologics currently under investigation for the treatment of atopic dermatitis include the IL-13 inhibitors lebrikizumab and tralokinumab [255], with the latter agent now approved in Europe. Another target is IL-31, for which the antibody nemolizumab is currently under investigation for the treatment of moderate-to-severe atopic dermatitis [256]. In addition, several clinical trials are addressing the potential of biologics that block IL-33 (etokimab) and Th22/IL-22 (fezakinumab) signalling pathways or target TNF Receptor Superfamily Member 4 (TNFRSF4, OX40) with ligands that prevent differentiation of naïve CD4-positive cells into inflammatory Th2 memory cells (for review see [257]). A further therapeutic strategy is the use of crisaborole, a topical anti-inflammatory phosphodiesterase-4 (PDE4) inhibitor approved by the US Food and Drug Administration (FDA) for the treatment of atopic dermatitis [258], which is superior even to topical immunomodulators such as tacrolimus and pimecrolimus [259]. Since the Janus kinase/signal transducers and activators of transcription (JAK/STAT) signalling pathway is associated with the development of atopic dermatitis, inhibitors of this pathway have been discovered as a possible pharmacotherapeutic option, of which baricitinib has been approved by the EMA for moderate-to-severe atopic dermatitis in adult patients (for review see [254]). A small molecule JAK1 inhibitor approved by the EMA for the treatment of moderate-to-severe atopic dermatitis in patients 12 years of age and older is upadacitinib, which recently showed a faster onset of action than dupilumab in a phase III study [260]. Furthermore, recently, the topical JAK inhibitor delgotinib was approved in Japan for the treatment of atopic dermatitis [261] and is currently in clinical trials for the European market [262]. In addition, combinations of immunomodulators and H_1_ antihistamines have been widely used to relieve pruritus in patients with moderate-to-severe atopic dermatitis [263].

#### 4.2.2. Preclinical Findings on the Effect of Cannabinoids in Atopic Dermatitis

In the context of hypersensitive skin reactions, a study using a mouse model of Th2-type contact hypersensitivity with pathophysiological features of mouse atopic-like dermatitis found that animals in which the CB_1_ receptor was knocked out showed a hypersensitivity reaction as measured by ear swelling, transepidermal water loss, Th2-type skin inflammatory reactions and serum IgE levels, all of which are appropriately characteristic of atopic dermatitis [264]. Furthermore, JTE-907 (Figure 1), a CB_2_ receptor inverse agonist, reduced itch-related responses in a murine model of atopic dermatitis to an extent comparable to that of tacrolimus [265]. In another work, the CB_2_ receptor agonist S-777469 showed an inhibitory effect on mite antigen-induced atopic dermatitis-like skin lesions in NC/Nga mice [5], with the latter exhibiting reduced eosinophil infiltration after treatment with S-77469. Collectively, these studies show conflicting results regarding the role of the CB_2_ receptor in atopic dermatitis, as both the CB_2_ receptor antagonist/inverse agonist JTE-907 and the CB_2_ agonist S-777469 produced positive effects. Thus, the role of the CB_2_ receptor seems to be a complex one here.

As a further mechanism of action one investigation found AEA and THC at submicromolar concentration were able to inhibit CGRP release from isolated rat and mouse skin preparations in a CB_1_ receptor-dependent way [266]. In addition, AEA and the newly synthesised CB_1_ agonists α-oleoyl oleoylamine ethanolamine (α-OOE; Figure 1) and α-OOS (Figure 1) suppressed mast cell proliferation, inhibited the release of inflammatory mediators and prevented the increase of skin fold thickness in mice with oxazolone-induced atopic dermatitis mice. These findings thus point to a crucial role of the CB_1_ receptor in modulating antigen-dependent IgE-mediated mast cell activation [267]. In another study, the symptoms of oxazolone-induced atopic dermatitis were inhibited by α-OOS [128], as evidenced by α-OOS-reduced transepidermal water loss, skinfold thickness and skin surface pH, while skin capacitance was increased. Also worth mentioning in this context is a recent prospective, randomised, double-blind, placebo-controlled study in dogs with atopic dermatitis in which a mixed oil based on CBD and CBD acid was tested [268]. No effect of treatment on IL-6, -8, -31, -34 and MCP-2 serum levels or skin lesions could be detected, although an improvement in itching was observed.

### 4.3. Allergic Contact Dermatitis

#### 4.3.1. Current Therapies of Allergic Contact Dermatitis

Allergic contact dermatitis is a type IV hypersensitivity reaction triggered by contact allergens. The impairment of the skin barrier here leads to increased permeability and penetration of allergens and triggers innate signalling pathways required for the activation of the adaptive T-cell response during the sensitisation phase, which is accompanied by the release of proinflammatory cytokines [269]. Although a recent review referred contact dermatitis as a “great imitator” that mimic many skin diseases such as atopic dermatitis, lichen planus and angioedema [270], a recent meta-analysis found no overall association between atopic dermatitis and contact sensitisation [271]. Concerning therapeutic measures, suspected allergens or cross-allergens should be avoided. In addition, topical glucocorticoids are the primary basis for treatment. Topical calcineurin inhibitors are indicated for prolonged use [270]. In contact dermatitis of the hand, which has an overall prevalence of 5.2% in men and 10.6% in women in Northern Europe [272], retinoids such as acitretin are an option for chronic symptoms [273]. It has also been shown that contact dermatitis of the hand can be treated long-term with oral cyclosporine and azathioprine [270], although the latter has only been tested in patients with the rare chronic actinic dermatitis [274].

#### 4.3.2. Preclinical Findings on the Effect of Cannabinoids in Allergic Contact Dermatitis

The crucial role of cannabinoid receptors in contact dermatitis was shown in animal models of DNFB-induced ear inflammation in C57BL/6J mice serving as a model of cutaneous allergic contact hypersensitivity, in which a cannabinoid receptor double knockout induced greater sensitivity to inflammatory conditions accompanied by increased expression of MCP-2 [3]. Cannabinoid receptor activation here decreased Toll-like receptor 3 (TLR3) ligand polyinosinic polycytidylic acid (poly-(I:C))-induced MCP-2 expression in the keratinocyte cell line HaCaT. Stimulation with poly-(I:C) serves as an in vitro model for contact dermatitis in this context. In addition, the latter study demonstrated a reduced allergic response after DNFB exposure in FAAH knockout mice. In another investigation, the CB_2_ receptor agonist S-777469 was shown to exert an inhibitory effect on DNFB-induced ear inflammation in dermatitis of Balb/c mice [5]. A contribution of TRPV1 to the anti-inflammatory properties of PEA was discovered, as PEA inhibits DNFB-induced ear inflammation in mice via a TRPV1-dependent mechanism [275]. The same group later found that CBD decreases MCP-2, IL-6, IL-8 and TNF-α via CB_2_ and TRPV1 in human keratinocytes stimulated with poly-(I:C) [276]. The latter investigation further reported other cannabinoids such as CBC, CBG, THCV and CBGV to inhibit MCP-2, IL-6 and IL-8. Further work found that PEA reduced the number of ear scratches in mice exposed to DNFB, which was abolished by CB_2_ and PPARα antagonists [277]. In this context, it should be mentioned that the rationale for the use of PEA in diseases such as allergic contact dermatitis lies not only in its endocannabinoid effect, but also in the fact that it is an essential component of the stratum corneum, which accordingly compensates for deficiencies when applied topically in diseased skin [278]. Finally, in a recent work, newly developed CB_2_ receptor agonists were tested for their symptom-reducing properties in atopic contact dermatitis. Here, in agreement with the previously shown data, the CB_2_ receptor full agonist “8g” was also demonstrated to reduce poly-(I:C)-induced MCP-2 expression in HaCaT cells [279].

In contrast to the beneficial effects of CB_2_ receptor activation described above, another study found that the CB_2_ receptor antagonist SR144528 inhibited the expression of pro-inflammatory cytokines, reduced the recruitment of eosinophils and attenuated ear swelling in a mouse model of oxazolone-induced contact dermatitis [171]. Consistent with this, the CB_2_ receptor inverse agonist JTE-907 showed anti-inflammatory properties in the carrageenin-induced paw oedema test in mice [280] and inhibited DNFB-induced ear swelling of mice [281,282].

Just as in the case of atopic dermatitis presented above, it is difficult to assess the extent to which activation or inhibition of the CB_2_ receptor brings perspective benefits for patients with allergic contact dermatitis. Particularly difficult in this context are findings that show on one occasion that CB_2_ knockout mice exhibit greater ear swelling in the DNFB-induced model of allergic contact dermatitis [3] and on another occasion that the CB_2_ knockout mice show less ear swelling in an IgE-dependent three-phase cutaneous response in the mouse ear [282]. The authors of the first mentioned study therefore suggested that CB_2_ antagonism may be beneficial initially but detrimental with chronic blockade [3].

An important role in skin homeostasis has likewise been evidenced for the cannabinoid target PPARα, which is a major trigger of skin inflammation [283]. In this context, PPARα-deficient mice were reported to exhibit greater epidermal thickening and increased recruitment of inflammatory cells from the skin compared to their wild-type counterparts [182]. Consistent with these results and the above-mentioned functional role of PPARα in the beneficial effect of PEA on atopic contact dermatitis [277], inhibition of endogenous PEA degradation was shown to improve symptoms in the DNFB-induced murine dermatitis model via PPARα [284]. Application of the NAAA inhibitor ARN077, which resulted in a large increase in PEA, caused a reduction in pathological skin thickening, scratching behaviour, a reduction in circulating IL-4, IL-5 and IgE levels, and an increase in IFN-γ. A comparable reduction in skin thickening by ARN077 could no longer be observed in PPARα knockout animals. Finally, the crucial role of NAAA was demonstrated in this study by the fact that DNFB-treated NAAA knockout mice showed significantly less skin thickening and greatly reduced scratching behaviour compared to wild-type littermates.

Finally, topical *N*-palmitoyl serinol, previously described as a CB_1_ agonist [132], was found to improve the epidermal permeability barrier and partially counteract transepidermal water loss and reduced hydration of the stratum corneum in DNFB-treated mice [285]. However, the latter study only speculated on a possible role of cannabinoid receptors in the *N*-palmitoyl serinol effect presented, without investigating it experimentally.

### 4.4. Psoriasis

#### 4.4.1. Current Therapies of Psoriasis

Psoriasis is an immune cell-mediated systemic inflammation that occurs in relapses and is characterised by recurrent exacerbations and remissions of thickened, erythematous and scaly plaques and other comorbidities [286]. An essential feature of psoriasis is the proliferation and keratinisation of epidermal cells (for review see [287]). Dendritic cells here over-secrete TNF-α and IL-23, leading to the differentiation of naïve T cells into the Th17 subtype, which under healthy conditions triggers the maintenance and recruitment of neutrophils for immune defence [288]. These cells exhibit increased production of IL-17 and IL-22. As a result, TNF-α and IL-17 cause epidermal hyperplasia and increased recruitment of inflammatory cells such as neutrophils. In addition, IL-17 increases the production of the proinflammatory cytokines IL-6 and IL-8 in keratinocytes [289].

The pharmacotherapy of psoriasis has some similarities with the treatment of atopic dermatitis, e.g., the use of immunosuppressants such as calcineurin inhibitors for mild-to-moderate facial psoriasis and intertriginous psoriasis [290] as well as topical corticosteroids such as betamethasone [291], which not only have an anti-inflammatory effect but also decrease keratinocyte proliferation and induce immunosuppression [292]. However, a whole range of small molecules and biologics have been approved specifically for different types of psoriasis.

Among topical treatments for psoriasis, there are several options for mild-to-moderate forms such as the use of dithranol, also known as anthralin, which inhibits epidermal hyperproliferation and the number of neutrophils in the epidermis and dermis [293]. Other recommended drugs for mild-to-moderate plaque psoriasis include vitamin D analogs such as calcipotriol, tacalcitol, maxacalcitol, paricalcitol and becocalcidiol [286]. One group of drugs that is still controversial in this context because of their side effects are the retinoids that are also used to treat acne, such as topically applied tazarotene [294]. Even more critical are systemically administered retinoids such as acitretin, which are recommended for psoriasis because retinoid acid receptor activation reduces STAT signalling, cell proliferation and epidermal differentiation, and inhibits scaling and thickness of psoriatic plaques [295]. Acitretin is recommended for treating moderate-to-severe psoriasis, but is clinically limited by a number of adverse side effects [296]. Other systemic treatment options include immunosuppressive therapies with methotrexate and cyclosporine A [286]. Although the mechanism of action is still unclear, fumaric acid esters such as dimethyl fumarate are used in Europe for treatment of moderate-to-severe psoriasis [297]. In addition, the treatment of moderate-to-severe psoriasis shares further common features with the treatment of atopic dermatitis, such as the use of PDE4 inhibitors like apremilast, which is only approved for psoriasis in Europe, and crisaborole, which is only approved for atopic dermatitis in Europe [298], as well as JAK inhibitors [299] and various immunosuppressants [300,301].

Finally, there are currently a plethora of biologics that can interrupt the pathophysiological cascade of advanced psoriasis. These include some biologics approved for the treatment of rheumatic diseases, such as the TNF-α blockers adalimumab, etanercept, infliximab and certolizumab [302]. Other treatment options for psoriasis are biologics that target IL-23, the key factor that triggers the activation of the Th17 inflammatory pathway. IL-23 consists of a p19 subunit linked to a p40 subunit, sharing it with IL-12, which triggers Th1 differentiation and proliferation. Therefore, antibodies targeting these factors have emerged as a treatment option for psoriasis. A currently approved antibody against the p40 subunit of IL-23/IL-12 is ustekinumab [303]. Other biologics for the treatment of psoriasis include antibodies against the p19 subunit of IL-23 such as guselkumab [304], tildrakizumab [305] and risankizumab [306]. An alternative option for the treatment of psoriasis is antibodies that directly interfere with the downstream Th17 pathway triggered by IL-23, resulting in increased release of IL-17A, -17F, -22, -26 and -29. A further relevant biologic in this context is ixekizumab, a humanised IgG4 monoclonal antibody that binds with high affinity to IL-17A/F heterodimers, thereby inhibiting the inflammatory processes involved in psoriasis [307]. Other IL-17-directed antibodies are secukinumab [308] and brodalumab, both of which bind to IL-17A [309].

Thus, even in the treatment of psoriasis, the newer substances are less small molecules and more biologics that act on the immune system and inflammatory markers.

#### 4.4.2. Preclinical Findings on the Effect of Cannabinoids in Psoriasis

An important factor and reason for the use of cannabinoids in psoriatic skin is their property to inhibit the proliferation of keratinocytes. This antiproliferative effect on keratinocytes was observed for the CB_2_ agonist JWH-015 and the compound BML190 [6], which has been described as an inverse agonist at the CB_2_ receptor [310,311,312]. A comparable antiproliferative effect was reported for the non-selective cannabinoid receptor agonist HU-210, in whose actions, however, cannabinoid receptors did not play a mediating role [6]. Instead, the authors of the study suspected PPARγ involvement in the described effect. Experiments with cultured normal human epidermal keratinocytes from human skin samples and HaCaT cells revealed that AEA sequentially initiates a signalling mechanism leading to activation of CB_1_, TRPV1, calcium influx and finally cell death, thereby regulating epidermal homeostasis, which in turn prevents the initiation or exacerbation of chronic hyperproliferative, pruritic and/or proinflammatory skin diseases [313]. In another study, using CB_2_ knockout mice, the CB_2_ receptor was shown to play an important protective role in imiquimod-induced psoriasis through immunosuppression, mainly by regulating the proliferation and differentiation of CD4-positive T cells and inhibiting the expression of cytokines and chemokines [178]. Accordingly, in these animal studies, symptom exacerbation was observed in CB_2_ knockout mice and in animals treated with the CB_2_ antagonist AM-630, while symptom relief was recorded in animals treated with the CB_2_ agonist JWH-133. In another report, AEA was noted to reduce the proinflammatory T-cell response via CB_1_ receptor-induced upregulation of the mammalian target of rapamycin (mTOR) inhibitor FK506-binding protein (FKBP)12, leading to downstream inhibition of the release of IL-12 and IL-23 from keratinocytes, which results in reduced Th1 and Th17 polarisation intercellularly [314]. These CB_1_ receptor-dependent regulations correspond to the regulatory profile required for the treatment of psoriasis.

In a recent report, CBD was found to reduce redox imbalance in UV-irradiated keratinocytes isolated from human skin tissue by decreasing ROS levels, increasing vitamin A and E, and specifically increasing the efficiency of the thioredoxin reductase system. Thioredoxin reductase is an enzyme whose activity is increased in keratinocytes from psoriasis patients. In this context, CBD increased thioredoxin reductase activity in keratinocytes from healthy individuals but decreased it in keratinocytes from psoriasis patients and UVB-irradiated samples [7]. In another recent publication, the lipidomic profile of keratinocytes from healthy individuals and patients with psoriasis was studied with and without UVB light in the presence and absence of CBD [315]. The study found that in the keratinocytes of patients with psoriasis, phosphatidylcholines, phosphatidylinositols, phosphatidylserines and ether-linked phosphoethanolamine are reduced and sphingomyelins are upregulated, while UVB, as used in UVB phototherapy, causes upregulation of phosphatidylcholine species, phosphatidylcholine plasmalogens and ether-linked phosphoethanolamine. The study revealed that CBD induced apoptosis in keratinocytes from psoriatic patients. However, in keratinocytes from patients irradiated with UVB, CBD enhanced the antioxidant properties and prevented the loss of transepidermal water. It is noteworthy that in the above studies, no experiments with receptor antagonists were performed to prove the causal involvement or non-involvement of receptors in the effects triggered by cannabinoids. However, Jarocka-Karpowicz et al. [7] showed that CBD increased AEA levels, upregulated CB_1_ receptors in UV-irradiated keratinocytes from healthy and psoriasis patients, increased CB_2_ receptors in keratinocytes from healthy and psoriasis patients and decreased CB_2_ expression in UV-irradiated keratinocytes from psoriasis patients. TRPV1 was also upregulated by CBD here, but only in keratinocytes that were not exposed to UV light. In addition, a massive accumulation of CBD in the membranes of the keratinocytes was found, which was further enhanced by UVB irradiation. Recently, CBD has also been reported to activate antioxidant metabolic pathways in keratinocytes and to modulate pathways involved in keratinocyte differentiation, skin development and epidermal cell differentiation, with the latter effect occuring via nuclear export and proteasomal degradation of the transcriptional repressor BTB Domain And CNC Homolog (BACH)1 [161]. However, in this study, topical CBD (0.1% or 1%) resulted in hyperproliferation of keratinocytes and wound healing of the skin of mice, indicating a harmful rather than beneficial effect of CBD on psoriasis.

### 4.5. Acne

#### 4.5.1. Current Therapies of Acne

Acne is a chronic inflammatory disease affecting the pilosebaceous unit. Symptoms include hyperseborrhoea due to increased androgen levels, inflammation and hyperkeratinisation of follicular keratinocytes [316]. The Global Burden of Disease Study identified acne vulgaris as the eighth most common skin disease, with an estimated worldwide prevalence (for all age groups) of 9.38% [317]. In the age group between 12 and 25 years, 85% of people in the United States develop acne [318]. Acne can occur non-inflammatory as an open or closed comedo, but also as an inflammatory form. Inflammatory forms of acne are usually papular or pustular acne caused by *Cutibacterium acnes* and are treated with, e.g., macrolides, clindamycin and tetracyclines (for review see [319]). In addition, *Staphylococcus epidermis* and *Malassezia furfur* can promote inflammation and induce follicular epidermal proliferation [320]. During puberty, sebum secretion is increased by dihydrotestosterone, which causes hyperproliferation of the follicular epidermis, leading to sebum retention [320]. Genetic variants associated with acne thus include genes that influence inflammatory responses, such as genes encoding TNF family proteins, and genes that influence sebaceous gland functions, particularly cytochrome p450 subtype 17A1 and the gene encoding follistatin [321].

Currently used therapies include topical and systemic options. Besides the antibiotics mentioned above, topical formulations contain retinoids such as retinoic acid, adapalene and tretinoin as comedolytic agents [322]. Azelaic acid is used as an antimicrobial and comedolytic substance that acts as a competitive inhibitor of 5α-reductase, thereby inhibiting the conversion of testosterone into 5α-dihydrotestosterone [323]. In addition, azelaic acid has the properties of an anti-skin-forming drug that inhibits the formation of comedones [324]. Topical salicylic acid is used for seborrhoea and comedonal acne and for pigmentation after acne has healed [325]. Consistent with its effect on sebocyte function, salicylic acid attenuates sebocyte lipogenesis by down-regulating the adenosine monophosphate-activated protein kinase (AMPK)/sterol response element-binding protein-1 (SREBP-1) pathway [326]. Another topical option is the use of dapsone, which was recently described as effective in a meta-analysis, with no significant difference in side effects compared with other drugs [327]. The reason why the safety of dapsone was questioned were reports showing that dapsone caused haemolytic anaemia in patients with glucose-6-phosphate dehydrogenase deficiency [328]. Systemic therapies further include isotretinoin [329]. Other options target hormone receptors with oral contraceptives containing low-dose oestrogen in combination with cyproterone acetate as antiandrogen [330]. Reduced production of androgens and inhibition of the effects of testosterone may eventually be achieved by spironolactone in male patients, as recently studied [331].

#### 4.5.2. Preclinical Findings on the Effect of Cannabinoids in Acne

Several studies have shown that cannabinoids affect sebocytes, especially their inflammatory properties, and could therefore be a therapeutic option for skin conditions such as acne. In this context, CBD has been reported to exert homeostatic and anti-inflammatory effects on sebocytes [332]. Here, the authors found that CBD inhibited lipogenic effects and sebocyte proliferation in vitro via activation of TRPV4 and mediated anti-inflammatory effects in an adenosine A2a receptor-dependent manner via upregulation of the endoplasmic reticulum stress-associated factor pseudokinase tribbles homolog 3 (TRIB3) and inhibition of NF-κB signalling. In another study, the same group investigated the phytocannabinoids CBC, CBDV, CBG, CBGV and THCV for their effects on human sebocyte functions. All of the above cannabinoids caused a decrease in lipopolysaccharide (LPS)-induced release of the proinflammatory cytokines IL-6 and IL-8 and a decrease in mRNA expression of IL-1α, IL-1β and TNF-α. Among these, CBC, CBDV and THCV inhibited arachidonic acid-induced lipogenesis and THCV also reduced sebocyte proliferation [333]. The anti-inflammatory properties of topically applied phytocannabinoids such as CBC, CBCV, CBD, CBDV, ∆^8^-THCV, Δ^8^-THC and THC were further confirmed in the Croton oil mouse ear dermatitis test, measured as inhibition of the oedematous response [334]. Moreover, preclinical studies revealed a possible anti-acne effect of hemp seed hexane extracts due to their anti-microbial, anti-lipogenic and collagen-promoting properties [335]. Finally, some studies have focused on the effect of cannabinoids against bacterial-induced inflammation. In this context, CBD was found to inhibit inflammation induced by extracellular vesicles of *Cutibacterium acnes* [336]. In line with this notion, CBD and CBG were found to inhibit the release of IL-8 and IL-1 from normal human epidermal keratinocytes exposed to *Cutibacterium acnes* [154]. Moreover, CBD was recently reported to have selective activity against a subset of Gram-negative bacteria [337]. Collectively, the antimicrobial effect of various cannabinoids may play a role in the effect on acne, which would need to be further investigated in future studies.

### 4.6. Systemic Sclerosis

#### 4.6.1. Current Therapies of Systemic Sclerosis

Systemic sclerosis, also called scleroderma, is a rare connective tissue disease with autoimmune character, which is mainly characterised by fibrosis of the skin, but also of the internal organs such as the lungs [338]. As the cannabinoid lenabasum has recently received orphan drug designation for the treatment of systemic sclerosis by the EMA [10], a more detailed description of the pathogenesis and pharmacotherapy of this disease is given here. Systemic sclerosis is characterised by fibroblast dysfunction with increased deposition of extracellular matrix proteins and small vessel vasculopathy leading to tissue hypoxia and autoantibody production [339]. Conventional therapy for scleroderma includes a variety of pharmacological interventions targeting a wide range of symptoms. These include, for example, vasoactive agents to control Raynaud’s disease as a typical symptom of scleroderma and to reduce the risk of amputation in the long term [340], as well as agents indicated to support healing, especially of digital ulcers, and to improve symptoms of pulmonary hypertension. Particularly difficult to treat are musculoskeletal damage, asthenia, pruritus and calcinosis, as well as skin fibrosis, whose pharmacological treatment options are currently limited to a few agents such as immunosuppressants [341]. Treatment of lung fibrosis with specific antifibrotic drugs is currently limited to blocking fibroblast growth factor receptor (FGFR) and platelet-derived growth factor (PDGF) and VEGF receptors by nintedanib [342], a triple angiokinase inhibitor [343]. Another critical symptom is the sclerodermal renal crisis, which is treated with angiotensin-converting enzyme (ACE) inhibitors [344]. As systemic sclerosis also affects internal organs, the therapy goes far beyond the conventional therapies of skin diseases with, e.g., cytostatics, anti-inflammatory substances and immunomodulators, which is why a complete description of the pharmacotherapy of systemic sclerosis is omitted here. However, the therapy of skin symptoms is similar to that of other inflammatory-immunological skin diseases. Topical therapies include tacrolimus ointments, potent corticosteroids and the vitamin D derivative calcipotriol. Systemic pharmacotherapies involve corticosteroids, but also mycophenolate mofetil, azathioprin, methotrexate, cyclophosphamide, rituximab as B-cell depletion therapy, nintedanib as a tyrosine kinase inhibitor, tocilizumab as an anti-IL-6 therapy and abatacept as an anti-cytotoxic T-cell lymphocyte-associated inhibitor (CTLA) 4 therapy [338]. The current challenge in systemic sclerosis is the treatment of skin fibrosis and other pathologies for which there is still no effective therapy, as recently noted [345].

#### 4.6.2. Preclinical Findings on Effects of Cannabinoids in Skin Fibrosis/Systemic Sclerosis

In the following chapter, bleomycin-induced fibrosis is listed at various points as a model system for systemic sclerosis, which in this context is a suitable model for the transfer of knowledge from animals to humans, although in its original form it is also an extended model for pulmonary fibrosis [346].

In an attempt to evaluate the effects of cannabinoids on fibrosis in an in vivo model, a corresponding effect was investigated in a mouse model of diffuse systemic sclerosis by injections of hypochlorite, which induced characteristic skin and lung fibrosis [347]. Here, the application of the non-selective cannabinoid agonist WIN 55,212-2 or the selective CB_2_ agonist JWH-133 reduced skin and lung fibrosis as well as fibroblast proliferation and the development of autoantibodies such as anti-DNA topoisomerase 1. The antifibrotic effect of WIN 55,212-2 was confirmed in the mouse model of bleomycin-induced systemic sclerosis [348]. Consistent with these findings, CB_2_ knockout mice exhibited increased skin thickness and collagen content in the skin and lungs, as well as increased proliferation of fibroblasts, suggesting that the CB_2_ receptor mediates an endogenous antifibrotic effect in the skin and lungs [347]. In line with this, another study reported that CB_2_ knockout mice are more sensitive to bleomycin-induced skin fibrosis [349].

In a further investigation, the novel CBD aminoquinone VCE-004.8, a dual PPARγ and CB_2_ receptor agonist, was shown to confer antifibrotic properties, such as reduction of differentiation of mouse embryonic fibroblasts into myofibroblasts. This was demonstrated by a reduction in α-smooth muscle actin (α-SMA) and inhibition of skin thickness, collagen accumulation around blood vessels, dermal mast cell degranulation and macrophage infiltration in the mouse model of bleomycin-induced fibrosis [12,13]. Using the same experimental model, EHP-101, a lipid formulation of VCE-004.8, was shown to inhibit skin and lung fibrosis and downregulate the expression of several fibrosis and inflammatory markers [14]. In a subsequent study, again based on the same experimental system, the authors revealed relief of skin fibrosis, reduction in skin thickness, recovery of lipoatrophy, reduction in Ashcroft score and dermal collagen content, and prevention of collagen accumulation around vessels with daily oral administration of EHP-101 (20 mg/kg body weight) or lenabasum (5 mg/kg body weight) [15]. In general, EHP-101 and lenabasum decreased the expression of genes involved in the inflammatory and fibrotic markers of systemic sclerosis, but EHP-101 was additionally able to upregulate factors related to angiogenesis and vasculogenesis. The property of EHP-101 to inhibit fibrosis in various tissues of mice was later also confirmed in a model of angiotensin II-induced fibrosis [350]. In the bleomycin-induced skin fibrosis model, celastrol, a natural compound isolated from the traditional Chinese medicinal plant *Tripterygium wilfordii* Hook F with the properties of a CB_2_ receptor agonist, and WIN 55,212-2 were found to be effective against skin fibrosis [351]. Celastrol and WIN 55,212-2 reduced skin thickness and α-SMA, a biomarker for myofibroblasts that accumulates in the skin during systemic sclerosis, and decreased mRNA levels of several inflammatory and fibrosis markers such as TNF-α, collagen 1α and TGF-β. In general, celastrol was superior to WIN 55,212-2 in all inhibitions of systemic sclerosis markers. In addition, WIN 55,212-2 was reported to reduce the deposition of extracellular matrix in fibroblasts from patients with diffuse systemic sclerosis and to counteract their transdifferentiation into myofibroblasts and resistance to apoptosis as typical cellular abnormalties in systemic sclerosis [352]. These effects were not inhibited by cannabinoid receptor antagonists.

In one study, lenabasum was found to prevent skin fibrosis in a mouse model in which constitutively active TβRI was overexpressed [219]. Interestingly, the latter study was able to provide data showing that lenabasum reduces the amount of pro-collagen type I propeptide in the cell culture medium of fibroblasts from patients with diffuse cutaneous systemic sclerosis as a marker of collagen synthesis in a PPARγ-dependent manner. Notably, data from animal studies have shown that deletion of PPARγ leads to increased susceptibility to bleomycin-induced skin fibrosis, suggesting that PPARγ suppresses fibrogenesis [353].

The role of the CB_1_ receptor in fibrotic diseases appears to be more in mediating a profibrotic effect. Accordingly, CB_1_ receptor activation has been linked with the development of pulmonary fibrosis [354,355,356]. Consistent with these findings, silencing of FAAH was associated with increased skin fibrosis in a mouse model of bleomycin-induced fibrosis [357]. In this study, the CB_1_ antagonist AM-281 reversed the profibrotic effects of FAAH inhibition, while the CB_2_ antagonist AM-630 enhanced fibrosis, indicating an opposite unfavourable effect of the CB_1_ receptor and a favourable effect of the CB_2_ receptor. In another study, also using the bleomycin-induced fibrosis model, similar results were obtained with CB_1_ receptor knockout mice that did not develop fibrosis, whereas activation of CB_1_ with ACEA increased leukocyte infiltration and enhanced the fibrotic response to bleomycin [358]. In a recent report, the peripherally restricted CB_1_/inducible nitric oxide synthase (iNOS) hybrid inhibitor zevaquenabant (MRI-1867) was found to reduce bleomycin-induced fibrosis in mice deficient in the multi-drug resistance gene 1 (MDR1), which encodes the drug efflux transporter p-glycoprotein (P-gp), but not in mice with intact MDR1 expression [169]. Consistent with the beneficial role of the CB_2_ receptor, another recent investigation reported that a dermal formulation of the CB_2_ receptor agonist GP1a reduced fibrosis in a mouse model of excitional wound healing [109].

### 4.7. Dermatomyositis

#### 4.7.1. Current Therapy of Dermatomyositis

Dermatomyositis belongs to the idiopathic inflammatory myopathies. Symptoms of this disease include fatigue, reduced mobility, dysphagia, dysphonia, calcinosis and dyspnoea due to weakened oesophageal and respiratory muscles [359]. Dermatomyositis may be accompanied by interstitial lung disease and is then associated with increased mortality [360]. However, there are also clinically amyopathic dermatomyositis subgroups of patients with the typical skin features of dermatomyositis such as heliotrope rash, facial erythema, Gottron’s papules and nailfold capillary abnormalities [361]. Treatment of this type of dermatomyositis involves a regimen that starts with topical steroids and calcineurin inhibitors and, depending on the response to therapy, includes further pharmacotherapeutic measures such as the use of hydroxychloroquine, methotrexate, intravenous immunoglobulins and mycophenolate mofetil. In addition, JAK inhibitors such as tofacitinib and biologics such as rituximab are also used [362].

#### 4.7.2. Preclinical Findings on Effects of Cannabinoids in Dermatomyositis

Reference has already been made to the orphan drug designation of lenabasum in the treatment of dermatomyositis [9]. The substance, which acts as a CB_2_ receptor agonist, suppressed the production of TNF-α, IFN-α and IFN-β in PBMCs of dermatomyositis patients [363]. In the context of dermatomyositis, lenabasum was also able to inhibit the expression of IL-31 in skin lesions, a cytokine upregulated in dermatomyositis patients suffering from pruritus, and to downregulate the release of IL-31 into the supernatants of PBMCs obtained from dermatomyositis patients and activated by the Toll-like receptor (TLR) 9 agonist CpG oligodeoxynucleotide [364]. In a recent publication, extensive profiling of dermatomyositis skin was performed and lenabasum was found to downregulate CD4-positive T cells, IFN-β, IFN-γ, IL-31 and pathophysiological CB_2_ receptor expression, while a similar effect was not observed in healthy skin [8].

### 4.8. Epidermolysis Bullosa

#### 4.8.1. Current Therapies of Epidermolysis Bullosa

Epidermolysis bullosa comprises a group of rare hereditary skin diseases characterised by marked fragility of the skin, which is why the disease is also called butterfly wing disease. It is predominantly caused by dominant-negative mutations in the genes encoding the keratins 5 or 14 [365]. There are four subtypes, namely epidermolysis bullosa simplex, junctional epidermolysis bullosa, dystrophic epidermolysis bullosa and Kindler syndrome [366]. Treatment of epidermolysis bullosa is usually supportive with wound care and relief of itching or pain [367]. Experimental approaches to the treatment of epidermolysis bullosa include gene therapies, cell-based therapies and protein therapies with recombinant collagen VII [367]. Pharmacological options currently being investigated include diacerein, a prodrug that after activation inhibits the plasma membrane-bound IL-1-converting enzyme, which can inhibit blistering of the skin [368]. Other pharmacological options have been derived from the treatment of atopic dermatitis, such as the reduction of blister formation after treatment with the PDE4 inhibitor apremilast [369]. Benefits for epidermolysis bullosa patients have also been shown for antipruritic therapies with dupilumab [370]. There is a body of research and anecdotal evidence for many other therapies, but the treatment of epidermolysis bullosa will be a challenge for future generations, in which cannabinoids could play a role that should not be underestimated.

#### 4.8.2. Case Reports on Cannabinoid Effects on Epidermolysis Bullosa

In relation to epidermolysis bullosa, where cannabinoids have been applied topically, case reports and retrospective observations showed promising effects. For example, in one observational study, topical CBD oil was found to reduce the symptoms of epidermolysis bullosa in three cases (a 6-month-old boy, a 3-year-old girl and a 10-year-old boy), which was associated with less concomitant analgesic medication, fewer blisters and a shorter healing time for active blisters [22]. A recent case report further found that a CBD oil formulation improved pain scores in a 21-year-old woman suffering from recessive dystrophic epidermolysis bullosa [371]. Here, CBD was administered at a dose of 124 mg (3.1 mg/kg body weight) twice daily. According to another case report, a sublingually administered THC/CBD combination in three patients with epidermolysis bullosa (a 64-year-old woman, a 41-year-old man and a 36-year-old man) also led to an improvement in pain scores, a reduction in itching and a reduction in the pain medication taken [19]. In this study, patient 1 received sublingual doses of a cannabinoid-based oil that were stepwise increased to 2.5 mg CBD and 1.625 mg THC, four times daily. Patient 2 reached the maximum dose of 3 mg CBD and 1.95 mg THC four times daily. Patient 3 ingested dried flowers of the female cannabis plant by inhalation and only later ingested an unspecified amount of the cannabinoid oil.

The results of a recently published international anonymous cross-sectional study on the use of medical cannabinoids showed an improvement in pain with a simultaneous reduction in pain medication, as well as a reduction of pruritus and an improvement of wound healing effects in patients with epidermolysis bullosa who used cannabinoid-based medications [20]. In this study, 71 surveys of patients taking cannabinoid-based medicines were evaluated. Both THC- and CBD-containing products were used, as well as those containing only CBD or only THC. However, a recently registered phase II/III study on the efficacy and safety of a CBD cream in epidermolysis bullosa patients was withdrawn [372]. Some hope rests on the use of CBD in this context. As noted in a commentary related to the use of cannabinoids in epidermolysis bullosa, it would be time “to start planning robust studies in patients with epidermolysis bullosa”, as the effects of pain are generally under-recognised in people suffering from epidermolysis bullosa [373].

### 4.9. Pyoderma Gangrenosum

#### 4.9.1. Current Therapies of Pyoderma Gangrenosum

Pyoderma gangrenosum is a painful ulcerative dermatosis associated with an autoimmune dysfunction characterised by dysregulation of neutrophil function and contribution of the Th17 immune axis which most frequently affects the lower extremity and appears associated with other diseases such as inflammatory bowel disease (34–65%), polyarthritis (16–19%) and hematologic disorders (12–20%) [374]. As pyoderma gangrenosum is an immunological disease, pharmacotherapy has a similar pattern to atopic dermatitis and psoriasis. Accordingly, the pharmacological management includes topical corticosteroids and tacrolimus, systemic treatment with corticosteroids, cyclosporine A, tacrolimus, azathioprine, methotrexate, mycophenolate mofetil as well as targeted biological therapies with anti-TNF-α treatments such as infliximab, adalimumab, etanercept, and IL-12/23 treatment with ustekinumab (for review see [375]). Biological treatment options for pyoderma gangrenosum furthermore include strategies from rheumatic treatment such as targeting IL-1 with anakinra, canakinumab and gevokizumab. Further cytostatic drugs applied in treatment of pyoderma gangrenosum include systemic therapies with cyclophosphamide, sulfasalazine and chlorambucil as well as antibiotics such as minocycline. A therapeutic problem associated with pyoderma gangrenosum is the treatment of wound-related pain requiring, for example, topical opioids which are associated with reduced likelihood of healing in patients with chronic wounds [376].

#### 4.9.2. Case Report on Cannabinoid Effects on Pyoderma Gangrenosum

A prospective case series of pyoderma gangrenosum [21] reported improvements, particularly in pain management, observed in three patients (a 50-year-old woman, a 76-year-old man and a 60-year-old woman) treated with cannabinoids. In one case, the topical oil ARGYLE™ (THC 5 mg/mL + CBD 6 mg/mL; 1.0 mL applied daily to the wound bed) and in the other two cases, the oil Bedrolite™ (THC 7 mg/mL + CBD 9 mg/mL; 0.5 to 1.0 mL applied twice daily to the wound bed plus once to three times daily for breakthrough pain) was used. Here, the case report showed an analgesic effect of topical medicinal cannabis associated with reduced opioid consumption, suggesting that cannabinoid therapies may also be beneficial and useful as topical applications for patients with pain-related skin conditions.

### 4.10. Beneficial Effects of Cannabinoids in Other Disorders

#### 4.10.1. Acute Inflammation

In an in vivo assay to identify cannabinoid substances that have an inhibitory effect on TPA-induced inflammation, a model of acute inflammation with erythema, edema and polymorphonuclear leukocyte infiltration [377], a series of 23 different analogues of synthetic cannabinoids were tested [378]. Among them, the topically applied naphthoylindoles JWH-018, -122 and -210 showed the strongest inhibitory effect on the formation of oedema on the ear of mice. In a recent in vivo study, it was also found that the topical formulation of CBD and PEA reduced TPA-induced ear oedema by 51.27% at 24 h and 65.69% at 48 h (in the control group, the reduction was 26.32% at 24 h and 44.21% at 48 h), suggesting that such a combined formulation has a remarkable effect on inflamed skin tissue [379]. These result are further supported by in vitro studies using a novel selective CB_2_ agonist, the 1,8-naphthyridine-2(1H)-one-3-carboxamide derivative JT11, that showed CB_2_ receptor-dependent inhibition of LPS-induced p42/44 MAPK and NF-κB activation, as well as inhibition of LPS-induced release of TNF-α, IL-1β, IL-6 and IL-8 from human PBMCs, with the described effects partially inhibited by the CB_2_ antagonist SR144528 [380].

#### 4.10.2. Keratin Disorders

The results of experiments focusing on the influence of cannabinoids on keratin synthesis, as already mentioned in Section 3.2.8, suggest that the use of certain cannabinoids may be beneficial in the treatment of diseases associated with keratin dysregulation, as is the case with a genodermatosis such as pachyonychia congenita [381] or a rare disease such as cicatricial alopecia (for review see [382]). On this basis, there has already been speculation about the extent to which endocannabinoid analogues might be helpful in the treatment of pachyonychia congenita [160]. A hyperkeratosis disorder, epidermolytic ichthyosis, a rare genodermatosis caused by mutations in keratins 1 or 10, has a profile of keratin dysregulation that may be counter-regulated by cannabinoids, as recently shown in an image correlation describing that ACEA upregulates the expression of keratin 10 in human epidermis and decreases the expression of keratin 1 in human skin organ culture [383]. However, to confirm the assumption that cannabinoids could help in such diseases, clinical studies would have to provide clarity.

#### 4.10.3. Scars and Keloids

Other work suggests a possible topical application of cannabinoids for the prevention or treatment of hypertrophic scars or keloids. Accordingly, the CB_1_ receptor agonist ACEA significantly increased collagen deposition in primary cultures of adult human fibroblasts obtained from TGF-β-treated human abdominal skin, while the CB_1_ antagonist AM-251 decreased α-SMA expression, collagen content and fibroblast differentiation [176]. Under the same experimental conditions, the authors also found that the CB_2_ receptor agonist JWH-133 decreased α-SMA expression and collagen content in TGF-β-stimulated cells, but not in resting cells. On the other hand, the CB_2_ receptor antagonist AM-630 also decreased α-SMA expression and collagen content in both resting and TGF-β-stimulated cells, but at higher concentrations (≥3 µM) [177]. The authors concluded that the effects produced by JWH-133 show potential for the treatment of hypertrophic scars or keloids in humans.

## 5. Clinical Trials with Cannabinoids

The above preclinical data has led to numerous clinical studies on the possible effects of cannabinoids on skin diseases that have been conducted and are still being conducted, with no posted results to date. One advantage of cannabinoids seems to be that they are relatively safe. For example, topical administration of a CBD-enriched ointment has recently been shown to be safe and effective in patients with inflammatory skin conditions [384]. In this study, no irritant or allergic reactions were observed during treatment. However, as this was an anecdotal, retrospective study without a blinded vehicle control group, the observations made here on the effects of cannabinoids on symptom relief cannot be generalised. Another non-randomised controlled trial conducted in two centres in Australia and New Zealand, looking at the safety of the transdermal CBD formulation ZYN002 in the context of the treatment of developmental disorders and epileptic encephalopathies, concluded that this CBD gel was safe and well tolerated [385]. Measures of this trial also included skin examination. In addition, an ongoing study is investigating the pharmacokinetic and pharmacodynamic effects of 5 different topically applied CBD-containing products, in which cognitive performance, working memory and psychomotor performance are also being interrogated [386]. In the following, clinical studies on the effect of cannabinoids in various diseases are listed alphabetically according to the corresponding disease.

### 5.1. Acne Vulgaris

CBD has been repeatedly discussed as a remedy for acne due to its anti-inflammatory properties [387,388]. As a consequence of the preclinical results, BTX 1503, a transdermal gel formulation containing synthetic CBD, is currently under clinical evaluation for the treatment of moderate-to-severe acne vulgaris [389]. The results of this study have not yet been published. Interestingly, however, in a single-blind comparative study to investigate tolerability and reduction of sebum and erythema, healthy men were treated twice daily for 12 weeks with a cream containing 3% cannabis seed extract on one cheek, with a control cream applied to the other cheek. The authors reported a reduction in the sebum and redness content of the skin on the sides treated with cannabis seed extract, suggesting a possible use of this extract for the treatment of acne, seborrhoea, papules and pustules [390]. Expanding the therapeutic options for acne would be a valuable step in treatment, because the treatment of acne with isotretinoin, for example, is known to cause serious side effects such as hypertriglyceridaemia, hepatotoxicity and mood disorders, e.g., depression and psychosis [391,392], which is why the status of these drugs has repeatedly been critically questioned [393].

### 5.2. Acute Inflammation

In a recent study investigating the anti-inflammatory effect of lenabasum, healthy male volunteers were injected with *E. coli* killed by UV light into the dermis, where they induced a strong local infiltration of neutrophils. Thereby, lenabasum showed a similar anti-inflammatory efficacy to prednisolone in terms of inhibiting neutrophil infiltration [221]. Furthermore, using inflammatory exudate from the injection site, lenabasum was found to accelerate the resolution of the inflammation by inhibiting chemoattractant leukotriene B_4_ and antiphagocytic prostanoids (PGE_2_, PGF_2α_ and thromboxane B_2_), while maintaining levels of the vasodilatory prostacyclin and having only a modest effect on the regulation of cytokines/chemokines that trigger acute inflammation. Instead, the proresolving factors lipoxin A_4_ and B_4_ as well as resolvin D_1_ and D_3_ were upregulated. At the local site of acute inflammation, lenabasum caused a dose-dependent increase in microvascular hyperaemia, which, however, was not oedematous, painful or inflammatory and was not accompanied by an increase in neutrophils or macrophages or increased biosynthesis of nitric oxide. The authors concluded that this effect was due to an aspirin-like action based on inhibition of vasoconstrictive thromboxane while maintaining prostacyclin levels.

### 5.3. Androgenetic Alopecia

A recent study found that daily application of a topical hemp oil formulation containing 3–4 mg of CBD and minimal amounts of other cannabinoids over a six-month period reduced hair loss associated with androgenetic alopecia [394]. The authors of the study announced that they will conduct a follow-up study with topical hemp oil extracts that contain high levels of THCV, CBDV and CBD. This study is now registered [395] and is also investigating, as a case series with 40 participants, the effect of two dosages (500 mg or 1000 mg of hemp oil per dispenser, which then results in either 1.7 mg or 3.4 mg of hemp extract per day) on androgenetic alopecia. Also worth mentioning is a case report of a 52-year-old man with alopecia areata showing that the application of cannabis oil in the area of hair loss helped the hair to grow again [396].

### 5.4. Asteatotic Eczema

A skin condition for which cannabinoids have also been tested is asteatotic eczema, also known as eczema craquelé or xerotic eczema. This is an itchy dermatitis with dry, scaly, inflamed skin that becomes cracked and fissured in advanced stages of the disease [397] and is the most common eczema of older people [398]. Treatment of asteatotic eczema consists of hydrating the skin through the use of lotions with high oil content. Pharmacological interventions are limited to topical corticosteroids such as fluocinolone, triamcinolone and betamethasone or topical pimecrolimus [399].

A monocentric randomised controlled trial concluded that regular use of a topical PEA/AEA emollient in patients with asteatotic eczema can improve both physicochemical and neuroimmuno-endocrine skin functions [400]. Symptoms improved by the PEA/AEA emollient included an increase in the 5 Hz current perception threshold and a reduction of itch, as well as improvements in skin surface hydration and transepidermal water loss, although the latter did not reach statistical significance compared with the PEA/AEA-free emollient.

### 5.5. Atopic Dermatitis

Two studies published in 2007 focused on the effect of PEA and adelmidrol in the treatment of atopic dermatitis [17,401]. The result of a study with 25 children and 18 adults was that a cream containing PEA caused a faster healing of a neurodermatitis flare-up on the respective treated side of the body and prolonged the mean time until the occurrence of another flare [17]. In another pilot study of 20 patients with mild atopic dermatitis, 2% adelmidrol caused improvement of erythema and pruritus in 60% of patients after 10–15 days, with complete resolution observed in 80% of patients after 4 weeks of treatment [401]. In a recent study on the effects of CBD (BTX 1204) on atopic dermatitis [16], CBD did not meet primary endpoints [402]. A more recent investigation confirmed the lack of effect of CBD but showed that JW-100, a novel CBD and aspartame formulation, resulted in significant improvements in atopic dermatitis after 14 days of topical application [18]. Patients with mild-to-moderate atopic dermatitis benefited particularly, as half of them had no or almost no symptoms after treatment. The authors confirmed here the effects that had previously been observed in a pilot study with a smaller group of patients [403]. In the latter study, 67% of the 16 patients who completed the study reported a decrease in itching and 50% noted an improvement in eczema of more than 60%. In another work analysing the effects and ingredients of hemp seed oil, patients with atopic dermatitis reported a decrease in skin dryness and itching, as well as a reduction in skin medication after using hemp seed oil [404]. In this study, however, the authors concluded that this effect was due to the high content of polyunsaturated fatty acids in hemp seed oil rather than specific cannabinoid effects. The CB_2_ receptor agonist S-777469 is also being investigated for the treatment of atopic dermatitis [405]. This double-blind, randomised, placebo-controlled, phase Ib/IIa, multiple-dose study evaluated the safety, tolerability, pharmacokinetics and pharmacodynamics of S-777469 in patients with mild-to-moderate atopic dermatitis. As secondary outcome, a possible antipruritic and anti-inflammatory effect was assessed. However, the results of this study have not yet been published. Another single-blind clinical trial conducted in 20 healthy volunteers addressed a possible anti-inflammatory effect of CBG in a sodium lauryl sulphate-induced skin irritation model of contact dermatitis. CBG here showed improvement over placebo in reducing transepidermal water loss and redness [154].

### 5.6. Atopic Eczema

A multinational, multicentre, observational, non-controlled, prospective cohort study of 2456 patients with mild-to-moderate atopic eczema who used an emollient containing PEA (ATOPA study), initiated as early as 2004, found that this formulation resulted in significant relief of skin dryness, excoriation, lichenification, scaling, erythema and pruritus [406]. Notably, the latter study did not include a placebo control. Another investigation is currently being prepared to investigate the size of eczema and its severity under the influence of a topical cream containing 1.5% PEA traded as Levagen^®^+ [407].

### 5.7. Dermatomyositis

A double-blind, randomised, placebo-controlled phase II study investigated the safety, tolerability and efficacy of lenabasum in 22 patients with dermatomyositis [408]. As a result of this study, the authors observed a decrease in the Cutaneous Dermatomyositis Disease Area and Severity Index (CDASI) in the lenabasum-treated group compared to the placebo group, which was significant at day 113 [409]. The company has meanwhile announced the follow-up multicentre, randomised, double-blind, placebo-controlled phase III study “DETERMINE study of lenabasum for the treatment of dermatomyositis” to further investigate the efficacy and safety of lenabasum in dermatomyositis with 176 participants [410]. This study has already been officially completed, but the results have not yet been published. Initial results from this phase III double-blind study in dermatomyositis showed that although lenabasum did not meet the primary and secondary efficacy endpoints, there were significant beneficial effects in the Total Improvement Score (TIS) with enhancement in muscle strength in patients with muscle weakness and significant favourable outcome in the CDASI with reduction in rash in patients without muscle weakness [411]. Notably, lenabasum has been granted an orphan drug designation status by the EMA for the treatment of dermatomyositis [9].

### 5.8. Hidradenitis Suppurativa

Hidradenitis suppurativa is a chronic, recurrent, inflammatory skin disease in which hair follicles in apocrine gland areas of the body are inflamed and manifest as painful nodules, abscesses, fistulas and scarring [412]. Antibiotics such as topical clindamycin are the first-line therapies here. Other options include oral antibiotics, retinoids, dapsone, oral contraceptives, oral immunomodulators and anti-TNF-α therapy [413]. A randomised, double-blind, placebo-controlled phase Ⅱ study, apparently interrupted prematurely in the recruitment phase by the COVID-19 pandemic, was designed to investigate the efficacy and safety of a cannabis oil in the treatment of 40 patients with hidradenitis suppurativa [414]. The basis of this study is the analgesic effect of cannabinoids, which is associated with a reduction in CGRP release and is therefore also mentioned as such in a review article on topically applicable analgesics in the context of hidradenitis suppurativa [415].

### 5.9. Neurogenic Flare Reaction

An early investigation addressed the effect of the peripherally delivered cannabinoid agonist HU-210 on histamine-induced inflammatory and pruritic responses in human skin of healthy volunteers. To this end, HU-210 was administered by skin patch or dermal microdialysis. In this study, the effects of HU-210 on vasodilatation and pruritus were measured after administration of histamine by iontophoresis or microdialysis [416]. To evaluate protein plasma extravasation, simultaneous administration of HU-210 and histamine was performed by dermal microdialysis, whereas in the analysis of vasodilatation and pruritus HU-210 was applied via a patch. HU-210 decreased itch rating, local blood flow and axon reflex flare response, while protein plasma extravasation was enhanced by HU-210 after histamine application. The fact that itch was diminished without reducing plasma extravasation of proteins suggests, according to the authors, that the relief of itch is not due to an antihistaminergic property of HU-210.

### 5.10. Postherpetic Neuralgia

Postherpetic neuralgia is an adverse effect appearing in herpes zoster cases with burning pain which is difficult to treat. Pain treatment includes non-steroidal anti-inflammatory drugs, opioid analgesics, carbamazepine, gabapentin and pregabalin [417]. In an open-labeled observational study, 5 of 8 patients reported a mean pain reduction of 87.8% after use of a cream containing PEA [418].

### 5.11. Pruritus (Chronic)

The effects of PEA have also been investigated in patients with chronic pruritus [419]. This prospective, randomised, controlled, open-label, non-interventional study on dry skin-induced chronic pruritus also examined the antipruritic effect of PEA compared to a PEA-free body lotion that served as a vehicle control. In this study, the authors did not find a significant difference between the lotion with and without PEA in terms of improvement of pruritus and quality of life.

### 5.12. Pruritis (Uremic)

In one study, 21 patients with uremic pruritis due to end-stage renal failure were tested with a cream containing structured physiological lipids and endogenous cannabinoids (AEA and PEA; patented as Physiogel AI^®^ cream) applied twice daily for a period of three weeks. In 8 patients the itching was completely eliminated and in 17 patients a complete reduction of xerosis was observed [420]. However, in this study the PEA group was not compared with a placebo group and no information was given on the amounts of AEA and PEA administered.

### 5.13. Psoriasis

Unlike other diseases, there are no solid clinical studies in the literature that directly address the effect of cannabinoids on patients’ psoriatic skin symptoms. Only CBD was studied, but it could not relieve psoriatic arthritis pain compared to placebo [421]. Furthermore, a case report was published in which a 33-year-old patient with psoriasis after failure of therapeutic application of hydrocortisone and triamcinolone acetonide cream successfully used a combination of cannabinoid-containing applications, consisting of THC distillate cream with medium-chain triglyceride oil, beeswax and sunflower lecithin and THC soap infused with a scent-free hemp soap, and a hair oil containing THC distillate dissolved in jojoba oil. The symptoms were improved after 2 days, and this effect lasted permanently when the THC-containing products were used as needed [422]. A spontaneous, anecdotal, retrospective study showed that CBD ointment also improved the Psoriasis Area and Severity Index (PASI) at day 90 from baseline, although the study design excluded a comparison with placebo [384].

### 5.14. Scalp Inflammation (Scalp Psoriasis/Seborrhoeic Dermatitis)

A recently published study of 50 subjects with mild-to-moderate scalp psoriasis or seborrhoeic dermatitis found that a CBD-containing shampoo significantly reduced both the severity and symptoms of scalp inflammation within two weeks with excellent tolerability [423]. However, it must be mentioned that this study did not include a comparison or placebo control with, for example, a cannabinoid-free shampoo, which significantly affects the conclusions drawn from this study.

### 5.15. Systemic Sclerosis

Further clinical trials were conducted on the effect of lenabasum in diffuse cutaneous systemic sclerosis, where lenabasum exhibited antifibrotic and anti-inflammatory activity [424,425,426]. In addition to a well-tolerated safety trial in which dizziness was the most common adverse effect, treatment with lenabasum was associated with improvement in several efficacy assessments of overall disease, skin involvement and patient-reported function. Lenabasum has recently received orphan drug status for the treatment of systemic sclerosis [10].

### 5.16. Urticaria (Chronic Spontaneous)

A recently registered, single-centre, placebo-controlled, open-label phase IIa trial is designed to investigate the safety, tolerability and efficacy of CBD as a potential treatment for patients with chronic spontaneous urticaria [427], another skin disorder in which histamine and other proinflammatory mediators involved in pathogenesis are released. However, the results of this study are not yet available.

### 5.17. Venous Leg Ulcers

Finally, data from a recent small open-label trial suggest that cannabinoids are a therapeutic option for rapid wound closure of previously non-healing venous leg ulcers [126]. This study tested the wound healing properties of cannabinoids in 14 patients with ulcers due to chronic venous hypertension and existing venous insufficiency. The test preparations were VS-12 and VS-14, which are hyaluronic acid and aloe vera gel-based (VS-12) and liposomal-based formulations of a mixture of CBD, THC, quercetin, diosmin, hesperidin and β-caryophyllene, respectively, applied to the wound bed and periwound area. Completely epithelialised wound closure was achieved in 11 of 14 patients within a median of 34 days, which is optimistic for larger studies that are definitely needed on this topic. 

The clinical studies presented in chapter 5 for the individual indications are summarised in Table 3, differentiated for PEA, CBD and lenabasum. 

## 6. Conclusions

Based on the current publications, it can be summarised that cannabinoid compounds have great potential in the treatment of skin diseases, both as topical applications and as systemic medications. It can also be assumed that other effects of cannabinoids, such as the improvement of impaired sleep that has been outlined in a recent systematic review [429], could have a positive influence on the quality of life of patients with painful and itchy skin diseases who often also suffer from sleep disorders. However, this effect of cannabinoids has not yet been investigated in patients with such skin diseases.

Since there are indications of possible effects of cannabinoids in several skin diseases other than those studied in patients so far, it can be further assumed that the potential of cannabinoids is far from exhausted and that further indications will be tested in the coming years. Accordingly, preclinical studies of cannabinoid-mediated molecular biological effects and mechanisms suggest that they may be effective in conditions such as rosacea, actinic keratosis, epidermolytic ichthyosis, hypertrophic scars, keloids, pachyonychia congenita, cicatricial alopecia or hidradenitis suppurativa. Robust clinical trials are needed in these areas.

Since some studies have already shown that, depending on skin type and gender, partly opposite effects can occur due to cannabinoids, clinical studies should primarily include larger patient collectives that allow for corresponding subgroup analyses. Accurate and quantitatively robust analysis of the clinical effects of cannabinoids on skin conditions is also important in that many cannabinoid-based products are now freely available on the market and, accordingly, are also used by patients for various treatments, including skin conditions [430], which in turn may be associated with risks.

From the currently available data, it appears that cannabinoid receptor agonists not only have positive effects, but that in some cases such as androgenetic alopecia or fibrotic diseases, CB_1_ receptor antagonists rather than agonists are indicated. Activation of cannabinoid receptors also leads to increased sebum production in the skin and may therefore be detrimental to the development of acne. In special cases such as fibrosis and systemic sclerosis, on the other hand, dual CB_2_/PPARγ agents such as lenabasum are more suitable cannabinoids. In some diseases, such as atopic dermatitis, the role of cannabinoid receptors must also be critically evaluated due to conflicting data. Due to these complex effects of agonists and antagonists at different cannabinoid-sensitive receptors on the skin, it is accordingly important to further investigate the role of these receptors in basic research.

The difficulty for the future development of new cannabinoid-based medicines also arises from the fact that in many publications it unfortunately remains open which receptors mediate the corresponding effects or whether a specific cannabinoid effect or a purely physicochemical fatty acid effect is involved in the improvement of skin structure and symptom relief. For this reason, too, further preclinical basic research is important. Only a more comprehensive understanding of the role of individual receptors of the endocannabinoid system in skin diseases will allow the targeted use of specific cannabinoids or even their chemical modifications to provide the optimal medication for each disease.

What can be stated with certainty today is that a major advantage of cannabinoids is their interaction with a variety of receptors, which can accordingly lead to improvements in skin diseases via multimodal mechanisms of action. However, the findings published so far seem to be far from a comprehensive understanding of the role of the PPAR, TRP, GPR55 and GPR119 receptors in connection with the effects of cannabinoids on the skin. In this context, based on current data, CBD appears to have an exceptionally attractive interaction profile with various target structures that is pharmacotherapeutically useful for patients with a variety of skin diseases. In addition, CBD has a relatively safe risk-benefit ratio, although its possible harmful influence on psoriasis is controversial.

Finally, the possibility of intervening pharmacotherapeutically with active substances in systems whose target structures contribute to a natural homeostasis of skin functions is of great interest for the therapy of skin diseases. The endocannabinoid system clearly belongs to these systems.

## Figures and Tables

**Table 1:** Regulation of endocannabinoids and cannabinoid receptors in tissue, plasma or cells from biopsies of skin diseases.

Endocannabinoid/ Receptor	Disease	Regulation	Species	Cells/Tissue	Ref.
AEA	TPA-induced acute ear inflammation	↔	mouse	inflamed ear versus control ear	[170]
	oxazolone-induced contact dermatitis	↔	mouse	oxazolone-treated ear versus control ear	[171]
	DNFB-induced contact dermatitis	↑	mouse	DNFB-exposed ear samples	[3]
	psoriasis vulgaris and psoriatic arthritis	↑	human	human plasma	[172]
	psoriasis	↓	human	keratinocytes from patients	[7]
	bleomycin-induced skin fibrosis	↑	mouse	fibrotic versus control skin	[169]
2-AG	TPA-induced acute ear inflammation	↑	mouse	inflamed ear versus control ear	[170]
	oxazolone-induced contact dermatitis	↑	mouse	oxazolone-treated ear versus control ear	[171]
	DNFB-induced contact dermatitis	↑	mouse	DNFB-exposed ear samples	[3]
	mite antigen-induced dermatitis	↑	mouse	skin lesions versus healthy skin	[5]
	psoriasis vulgaris and psoriatic arthritis	↑	human	human plasma	[172]
	psoriasis	↓	human	keratinocytes from patients	[7]
	bleomycin-induced skin fibrosis	↑	mouse	fibrotic versus control skin	[169]
CB_1_	DNFB-induced contact dermatitis	↓	mouse	DNFB-exposed ear samples	[3]
	psoriatic arthritis	↑	human	granulocytes from patients	[172]
	psoriasis vulgaris	↔	human	granulocytes from patients	
	atopic dermatitis	↓	human	itchy lesional skin	[173]
	psoriasis	↓	human	itchy lesional skin	
	seborrheic keratosis	↓	human	seborrhoeic keratosis affected tissue	[156]
	streptozotocin-induced type 1 diabetes	↓	mouse	skin tissue	[166]
CB_2_	DNFB-induced contact dermatitis	↑	mouse	DNFB-exposed ear samples	[3]
	incised wounds	↑	mouse	various cell types	[175]
	psoriatic arthritis	↔	human	granulocytes from patients	[172]
	psoriasis vulgaris	↑	human	granulocytes from patients	
	atopic dermatitis	↓	human	itchy lesional skin	[173]
	psoriasis	↓	human	itchy lesional skin	
	dermatomyositis	↑	human	lesional skin and PBMCs	[8]
	imiquimod-induced psoriasis	↑	mouse	psoriatic skin lesion	[178]

↔, not regulated; ↑, upregulated; ↓, downregulated; AEA, *N*-arachidonoyl ethanolamine (anandamide); 2-AG, 2-arachidonoyl glycerol; CB_1_, cannabinoid receptor 1; CB_2_, cannabinoid receptor 2; DNFB, 1-fluoro-2,4-dinitrobenzene; PBMCs, peripheral blood mononuclear cells; TPA, 12-O-tetradecanoylphorbol-13-acetate.

**Table 2:** Regulation of cannabinoid-modulated receptors in human skin tissue biopsies or plasma.

Receptor	Disease	Regulation	Cells/Tissue	Ref.
TRPV1	erythematotelangiectatic rosacea	↑	affected skin	[179]
	phymatous rosacea	↑	affected skin	
	atopic dermatitis	↑	pruritic lesional skin	[173]
	psoriasis	↑	pruritic lesional skin	
TRPV2	erythematotelangiectatic rosacea	↑	affected skin	[179]
	papulopustular rosacea	↑	affected skin	
	phymatous rosacea	↓	affected skin	
	atopic dermatitis	↑	pruritic lesional skin	[173]
	psoriasis	↓	pruritic lesional skin	
TRPV3	erythematotelangiectatic rosacea	↑	affected skin	[179]
	papulopustular rosacea	↑	affected skin	
	phymatous rosacea	↑	affected skin	
	atopic dermatitis	↓	pruritic lesional skin	[173]
	psoriasis	↑	pruritic lesional skin	
TRPV4	phymatous rosacea	↑	affected skin	[179]
	chronic idiopathic pruritus	↑	pruritic skin	[180]
GPR55	psoriasis vulgaris, psoriatic arthritis	↑	granulocytes from patients	[172]
GPR119	acne	↓	sebaceous glands	[99]
PPARα	actinic keratosis	↓	affected skin	[181]
	lichen planopilaris	↔	lymphocytic scarring alopecias	[149]
	atopic dermatitis	↓	eczematous skin	[182]
	melasma	↓	affected skin	[183]
	psoriasis	↓	pruritic lesional skin	[173]
	systemic sclerosis	↓	fibrotic skin tissue	[184]
PPARγ	actinic keratosis	↔	affected skin	[181]
	lichen planopilaris	↓	lymphocytic scarring alopecias	[149]
	photoageing in human skin	↓	photoaged or acutely UV-irradiated human skin	[185]
	atopic dermatitis	↓	atopic lesions	[186]
	psoriasis	↓	psoriatic lesions	
	psoriasis	↓	pruritic lesional skin	[173]
	systemic lupus erythematosus	↓	circulating CD14+ monocytes	[187]
	systemic sclerosis	↓	lesional skin fibroblasts	[98]
	systemic sclerosis	↓	fibrotic skin tissue	[184]
PPARδ	psoriasis	↑	psoriatic lesions	[188]
	lichen planopilaris	↔	lymphocytic scarring alopecias	[149]

↔, not regulated; ↑, upregulated; ↓, downregulated; GPR, G protein-coupled receptor; PPAR, peroxisome proliferator-activated receptor; TRPV, transient receptor potential vanilloid.

**Table 3:** Clinical studies on the 3 most important compounds in the investigation of cannabinoid effects on patients’ skin.

Substance	Disease/ Condition	Study Design	Formulation	Outcome	Ref.
PEA	asteatotic eczema	randomised, monocentric, double-blind, comparative trial	Physiogel^®^ A.I. Cream ^1^; combination with AEA	higher capacitance of skin surface; improved skin barrier function; reduction of pruritus	[400]
	atopic dermatitis	randomised, investigator-blinded, split-body trial	Physiogel^®^ A.I. Cream ^1^	faster healing; reduction of flares	[17]
	atopic eczema	multinational, multicentre, observational, non-controlled, prospective cohort study	Physiogel^®^ A.I. Cream ^1^	reduction of skin dryness, excoriation, lichenification, scaling, erythema and pruritus	[406]
		randomised, interventional, double-blind trial	Levagen^®^+ ^2^	no results posted yet	[407]
	chronic pruritus	randomised, prospective, controlled, open-label, noninterventional study	Physiogel^®^ Calming Relief A.I. Body Lotion	no significant improvement of pruritus and quality of life	[419]
	postherpetic neuralgia	open observational study; no placebo control	Physiogel^®^ A.I. Cream ^1^	reduction of pain and pruritus	[418]
	uremic pruritis	preliminary observational study; no placebo control	Derma Membrane Structure^®^; combination with AEA	reduction of pruritus and xerosis	[420]
CBD	acne vulgaris	randomised, double-blind, placebo-controlled phase II study	BTX 1503 liquid formulation	no results posted yet	[389]
	andro- genetic alopecia	case series study	CBD-rich hemp oil extract	enhanced hair regrowth	[394]
	atopic dermatitis	randomised, double-blind, placebo-controlled, phase II study	BTX 1204 liquid formulation	no significant improvement	[16,402]
		double-blind, placebo-controlled interventional study	JW-100 (topical formulation containing CBD and aspartame)	improvement in ISGA ^3^ scale	[18]
		observational study	topical CBD	decrease in itch; improvement in eczema	[403]
	chronic spon- taneous urticaria	placebo-controlled, open- label, single center, phase IIa study	oral CBD	no results posted yet	[427]
	psoriatic arthritis	randomised, double-blind, placebo-controlled trial	oral CBD	pain reduction	[421]
	scalp psoriasis/ seborrheic dermatitis	observational study	REVITA.CBD^®^ SHAMPOO ^4^	reduction of arborizing vessels, twisted capillaries, scales, erythema, itching and burning	[423]
Lenabasum	dermato- myositis	randomised, single-center, double-blind, placebo-controlled phase II study	oral JBT-101	reduction of CDASI ^5^	[408,409]
		randomised, multicenter, double-blind, placebo-controlled, phase III study	oral JBT-101	reduction of CDASI ^5^ (patients without muscle weakness); improvement in TIS ^6^ (patients with muscle weakness)	[410,411]
	healthy volunteers ^7^	randomised, open-label, placebo-controlled study	oral anabasum	inhibition of neutrophil infiltration; resolution of inflammation	[221]
	systemic sclerosis	randomised, placebo- controlled phase II trial	oral lenabasum	improvement in CRISS ^8^	[424,425]

^1^: Cream containing Olea Europeaea, glycerol, pentylene glycol, palm glycerides, olus, hydrogenated lecithin, squalane, betaine, sarcosine, acetamide, hydroxyethylcellulose, sodium carbomer, carbomer and Xanthan Gum [406]; ^2^: Levagen^®^+ topical formulation contains PEA, coconut oil (fractionated), polyglycerol polyricinoleate, citrus oil, olive oil, lecithin, DL-α-tocopheryl acetate and silicon dioxid [428]; ^3^: Investigator’s Static Global Assessment (ISGA); ^4^: shampoo containing CBD, ketoconazole, rooibos tea, taurine, caffeine, apple polyphenol, cysteine, spin traps, carnitine tartrate, biotin, emu oil, methylsulfonylmethane and ornithine [423]; ^5^: Cutaneous Dermatomyositis Disease Area and Severity Index (CDASI); ^6^: Total Improvement Score (TIS); ^7^: Inflammation triggered by intradermal injection of UV-killed *E. coli* into the forearms of healthy volunteers; ^8^: Combined Response Index in Diffuse Cutaneous Systemic Sclerosis (CRISS).
